# Arbuscular Mycorrhizal Fungi Taxa Show Variable Patterns of Micro-Scale Dispersal in Prairie Restorations

**DOI:** 10.3389/fmicb.2022.827293

**Published:** 2022-07-22

**Authors:** Alice G. Tipton, Donald Nelsen, Liz Koziol, Eric B. Duell, Geoffrey House, Gail W. T. Wilson, Peggy A. Schultz, James D. Bever

**Affiliations:** ^1^Department of Biology, St. Louis University, St. Louis, MO, United States; ^2^Kansas Biological Survey and Center for Ecological Research, University of Kansas, Lawrence, KS, United States; ^3^Natural Resource Ecology and Management, Oklahoma State University, Stillwater, OK, United States; ^4^Department of Biology, Indiana University, Bloomington, IN, United States; ^5^NEON, Boulder, CO, United States; ^6^Environmental Studies Program, University of Kansas, Lawrence, KS, United States; ^7^Department of Ecology and Evolutionary Biology, University of Kansas, Lawrence, KS, United States

**Keywords:** dispersal, restoration ecology, grassland, inocula, plant–microbial interactions, arbuscular mycorrhizal fungi

## Abstract

Human land use disturbance is a major contributor to the loss of natural plant communities, and this is particularly true in areas used for agriculture, such as the Midwestern tallgrass prairies of the United States. Previous work has shown that arbuscular mycorrhizal fungi (AMF) additions can increase native plant survival and success in plant community restorations, but the dispersal of AMF in these systems is poorly understood. In this study, we examined the dispersal of AMF taxa inoculated into four tallgrass prairie restorations. At each site, we inoculated native plant species with greenhouse-cultured native AMF taxa or whole soil collected from a nearby unplowed prairie. We monitored AMF dispersal, AMF biomass, plant growth, and plant community composition, at different distances from inoculation. In two sites, we assessed the role of plant hosts in dispersal, by placing known AMF hosts in a “bridge” and “island” pattern on either side of the inoculation points. We found that AMF taxa differ in their dispersal ability, with some taxa spreading to 2-m in the first year and others remaining closer to the inoculation point. We also found evidence that AMF spread altered non-inoculated neighboring plant growth and community composition in certain sites. These results represent the most comprehensive attempt to date to evaluate AMF spread.

## Introduction

In recent decades, the role of plant–soil microbial interactions in structuring plant communities has gained attention in the field of ecology. Feedback between plants and the soil community can impact plant succession, invasion, community composition, and plant diversity (Van der Putten et al., 2013; Bever et al., 2015). The dynamics of plant–soil feedback can depend on the level of dispersal of both plant and soil organisms (Molofsky and Bever, 2002; Bever et al., 2012; Michaels et al., 2020). This is particularly true of mutualisms, in which local dispersal is associated with increased stability (Bever et al., 2009; Mack, 2012). Microbial dispersal limitation can influence biogeography (Delavaux et al., 2019, 2021) and succession and the speed of recovery post-disturbance (Middleton and Bever, 2012; Kardol et al., 2014; Bauer et al., 2015; Wubs et al., 2016).

In many degraded ecosystems, restoring soil microbial communities and plant–microbial interactions have been proposed as essential to re-establishing complete and diverse native plant communities (Koziol et al., 2018). Specifically, in grasslands, arbuscular mycorrhizal fungi (AMF) may play an important role in structuring plant communities. Late successional plant species are more likely to rely on AMF and show AMF species-specific growth responses compared to early successional native or non-native plant species (Koziol and Bever, 2015, 2016, 2017; Cheeke et al., 2019). However, AMF community composition in grasslands with a history of disturbance remains different from those of remnant prairies, even in cases when remnant undisturbed prairies are nearby (House and Bever, 2018; Tipton et al., 2018). Some rare AMF taxa remain absent in disturbed sites altogether, suggesting that some AMF species do not readily re-establish disturbed sites on their own (House and Bever, 2018; Tipton et al., 2018). The absence or decreasing abundance of certain AMF taxa could explain why in sites with a history of large soil disturbances, late-successional or rare plant species often do not re-establish, even with abundant seed addition (Martin et al., 2005; Polley et al., 2005).

Although dispersal mechanisms are well known for higher-order organisms (Clobert et al., 2012), AMF dispersal mechanisms are still poorly understood (Paz et al., 2020). Studies suggest that some AMF spores can disperse *via* wind, but most wind-dispersed spores are small spore species belonging to Glomeraceae, and survival of aerially dispersed spores can be low (Warner et al., 1987; Allen et al., 1989; Egan et al., 2014; Chaudhary et al., 2020). A few species of *Glomeromycota* produce specialized subterranean sporocarps that are attractive to rodents, which act as dispersal agents (Gehring et al., 2002; Mangan and Adler, 2002). All AMF can also disperse *via* hyphal spread in the soil, and for many taxa, this may be the primary mechanism of dispersal. The rate of spread through hyphal growth is not well characterized (Paz et al., 2020), but is likely dependent on the local environment. As AMF are obligate mutualists, compatible plant hosts are necessary for their survival and the availability of quality host roots will likely influence the hyphal rate of spread. However, although still not well characterized, some studies suggest that AM hyphae can spread out into the soil to at least 1-m distances with few plant hosts (Chaudhary et al., 2014). The rate of spread will also likely vary between AMF taxa, as AMF display different growth strategies, with some producing more or less external hyphae than others (Abbott and Robson, 1985; Friese and Allen, 1991), and vary in their response to particular host species (Bever et al., 1996). Therefore, certain AMF taxa may be able to explore soil at greater distances than others.

When native plant species in restored prairie sites are inoculated with AMF collected from the remnant prairie, benefits can extend to non-inoculated plants at least 2-m away from the inocula source within the first growing season (Middleton and Bever, 2012; Middleton et al., 2015). This suggests that AMF hyphae can move meters through the soil in one growing season. However, the identity of the AMF that has spread and whether particular taxonomic groups are more likely to spread is not known. Moreover, it is unclear whether these hyphae move *via* infections of new hosts, moving from one plant to the next, or if individual hyphae can spread long distances without intermediate plant hosts. Should they be moved through intermediate hosts, then host quality in the plant community would modify the rates of the AMF hyphal spread. For example, we can hypothesize that certain AMF species may spread faster and farther with appropriate hosts along the way. Alternatively, some AMF species may be able to spread long distances independent of the plant community composition.

In this study, we integrate field inoculation of nurse plants with environmental sequencing to evaluate the rate of spread of native AMF from points of inoculation into the surrounding plant community during restoration. We placed native AMF inocula along with native nurse plants into sites once dominated by non-native grass species. Using environmental sequencing, we tracked the spread of AMF taxa present in the inocula in the plant community to determine how far and quickly AMF spread from inoculation points into the soil. We tracked the spread of AMF in areas with and without established “bridges” of native plant hosts, to determine the role of host quality in AMF movement through the soil. We also measured changes in the plant community and relative abundance of AMF using phospholipid fatty acid (PLFA) analysis to determine how the spread of inoculated AMF may alter above- and below-ground communities over time.

## Materials and Methods

### Study System

We established prairie reconstruction experiments at three different field sites across the Midwest in both 2014 and 2015: Chanute Air Force Base near Rantoul, IL (Chanute, 40°28′76.96″N, −88°13′36.23″W, 2014), Ft. Riley Military Base in Ft. Riley, KS (Ft. Riley, 2014), and Tinker Air Force Base near Oklahoma City, OK (Tinker, 35°24′54.96″N 97°24′37.02″W, 2015). Chanute was dominated by C_3_ *Schedonorus arundinaceus*. At Ft. Riley, we established two different sites with two unique grass dominants: C_3_ *Bromus inermis* (Ft. Riley *B. inermis*) and C_4_ *Bothriochloa bladhii* (Ft. Riley *B. bladhii*). Tinker was dominated by C_4_ *Bothriochloa ischaemum*. Because of variation in dominant exotic invaders and other environmental variations between sites, we used different plant removal methods in each site before prairie planting (Supplementary Table S1), including disking, spraying 5% glyphosate, or installing black tarps (4.88 × 4.88 m) to solarize plots following the methods of Upadhyaya and Blackshaw (2007).

### Inocula

#### Whole Soil Inocula

To compare the effects of pure AMF inocula to inocula containing the full suite of soil microbes, we collected and created a “whole soil” treatment for our experiment. We collected whole soil inocula at remnant prairies ranging from 0 to 25 km from each field site (Koziol et al., 2021). At remnant prairies, we collected 0.5 L for each of the five randomized samples of field soil to a depth of approximately 10 cm. The soil was sieved through an 8 mm sieve and stored at 4°C before being used as inoculum. Both Ft. Riley sites received the same whole soil inocula. Sub-samples from each whole soil inocula were stored at−20°C for molecular identification.

#### AMF Inocula

AMF inocula used in this study contained AMF collected and cultured from remnant prairies. In 2012, we extracted spores from the field-collected remnant prairie soil from the same locations used for the whole soil treatments using the methods of Morton et al. (1993). AMF species were separated microscopically. We created single spore cultures using the methods of Koziol and Bever (2016) (See Supplementary Material). Before field inoculation, multiple spore cultures from each site were mixed to create three site-specific cultures for each of the restoration sites (Ft. Riley, Tinker, and Chanute). Sub-samples from each site-specific mixed culture were stored at −20°C for molecular identification.

### Experimental Design

**Figure 1:**
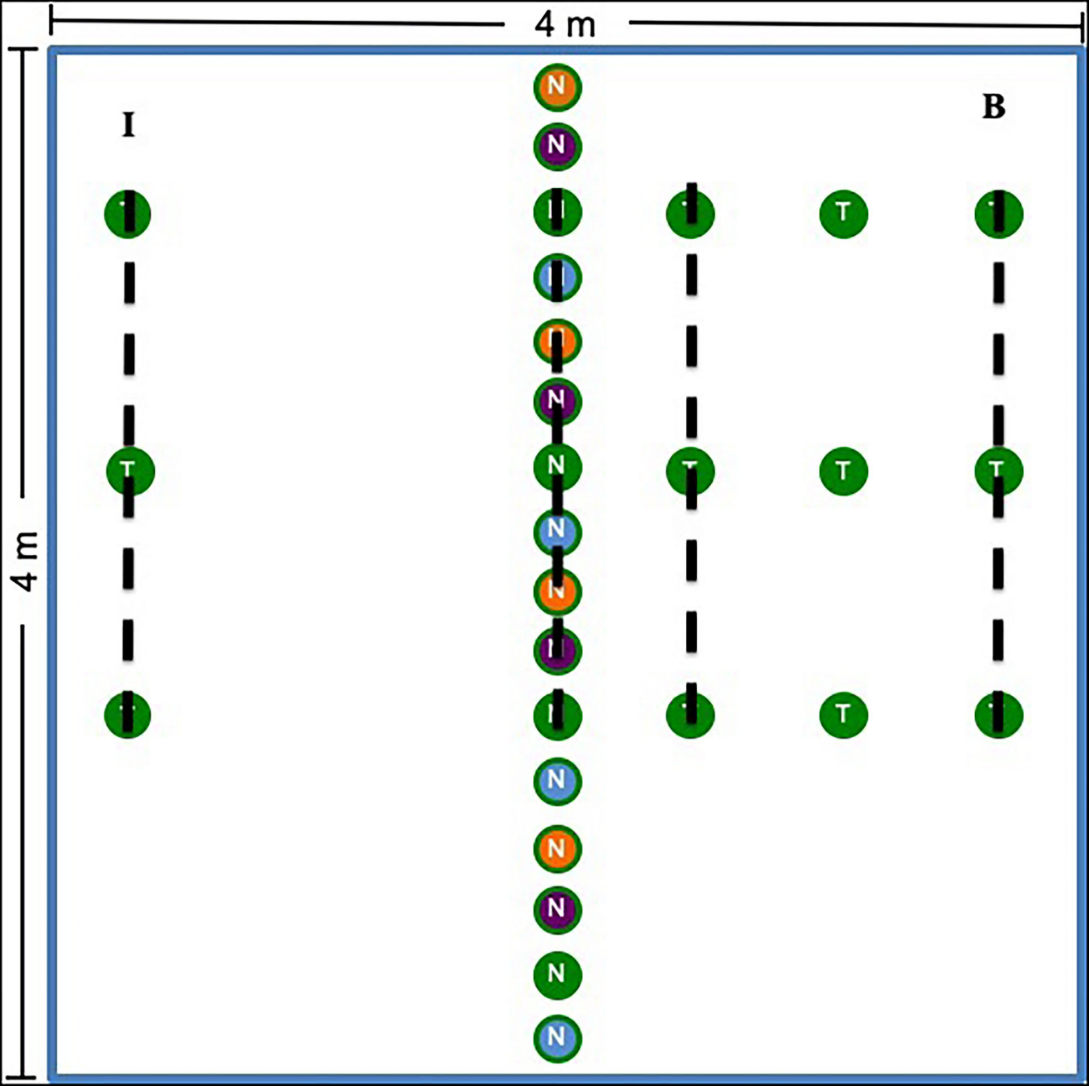
Plot design for the experiment. The center row represents the nurse plant row, which was either inoculated with whole prairie soil, prairie AMF only, or sterilized control soil (non-inoculated) depending on the plot treatment. The colors represent species. Four species were used as nurse plants. In Chanute and Tinker, test plants (*S. scoparium* at Chanute and *A. gerardii* at Tinker) were planted in rows 0.5-, 1-, and 2-m away from the nurse plant row. On one side, only the 2-m plants were added (I), whereas on the other side (B), plants were placed at 0.5-, 1-, and 2-m away from the nurse plant row. The dotted lines represent where soil and root samples were taken for molecular analysis. Plant community data were collected 0.5-, 1-, and 2-m away from the nurse plant row, and the side on which these data were taken was selected randomly each year and varied by site.

In May 2014 at Chanute and Ft. Riley and in May 2015 at Tinker, we assigned treatments to plots using a randomized block design. Chanute contained 9 blocks and 27 plots, while all other sites contained 7 blocks and 21 plots in total (69 plots total across all four sites). Each of the three plots in each spatially stratified block was randomly assigned one of the following treatments: nurse plants established with AMF inocula, nurse plants established with whole prairie soil inocula, or nurse plants established with sterilized control soil (hereafter referred to as non-inoculated). Because sites spread across a large rainfall and geographic gradient, different nurse plant species were used in various sites (Supplementary Table S1; Supplementary Methods S2 for nurse plant growth and inoculation). We planted 16 nurse plants in a row down the center of each of the 16-m^2^ plots (Figure 1). Each plot received four replicates of each nurse plant species of the corresponding treatment (16 nurse plants total). Replicates were repeated in the same order across each plot in each site. At Chanute and Tinker, we also planted grass test plants inoculated with sterilized control soil (non-inoculated) on either side of the nurse plant row (*Andropogon gerardii* in Tinker and *Schizachyrium scoparium* in Chanute). On one randomly selected side, we planted three test plants in three rows 0.5-m apart (bridge side, Figure 1). On the opposite side, we planted three test plants 2-m away from the nurse plants (island treatment).

### Soil and Root Sampling

Each growing season, we collected soil and root samples for molecular and phospholipid fatty acid (PLFA)/neutral lipid fatty acid (NLFA) analysis (Supplementary Table S2). In sites without test plants, we collected at the nurse plant row, 0.5-m away from the nurse plant row, 1.0-m away, and 2-m away from the nurse plant row. For Chanute and Tinker, we collected at the nurse plant row, 0.5-m away on the bridge side to collect near test plants, and 2-m away on both the island and bridge side (both years at Chanute and the second year at Tinker, Figure 1). We collected four 2-cm diameter samples approximately 10 cm deep using a soil core along each sampling row. Sampling was concentrated away from the plot edges. Soil corers were cleaned with 80% ethanol between each sampling row. Soil corers for each row were mixed and split into sub-samples fro molecular and PLFA/NLFA analysis.

### Molecular Analysis

Fresh roots from all 2014 samples and Chanute samples in 2015 were used for DNA extraction. For all other 2015 samples and inocula samples, the soil corers containing roots were frozen before extraction, and DNA was extracted from soil sample/root mixes. DNA was extracted from 0.025 g roots or 0.25 g of soil/roots using the Power Soil Kit (Qiagen, Carlsbad, CA) with a modified beat beating step. To amplify AMF-specific sequences of the large subunit (LSU), we conducted PCR with the primer pair LROR (Bunyard et al., 1994) and FLR2 (van Tuinen et al., 1998) amplifying an approximately 850-bp region. PCR amplification procedure was as follows: 94°C for 5 min; then 35 cycles of (1) 94°C for 30 s, (2) 48°C for 30 s, and (3) 72°C for 45 s; and ending with 72°C for 10 min. We purified PCR products with the AMPure XP bead system (Beckman Coulter, Indianapolis, IN). An equimolar amount from each sample was pooled and sequenced on the Illumina MiSeq platform to produce two non-overlapping 300 bp reads (Center for Genomics and Bioinformatics, Indiana University).

The resulting sequences were quality screened (quality score = 10) per read pair, and we removed chimeras using the –uchime_denovo function in VSEARCH (Rognes et al., 2016). Because individual AMF cells contain high levels of rDNA sequence variation (House et al., 2016) and we were interested in the movement of isolates rather than genetic variants within isolates, we analyzed OTUs rather than ASVs. We clustered the resulting sequences using AbundantOTU (Ye, 2010), using a 97% sequence similarity threshold. We then added and aligned the consensus OTU sequences to a reference alignment of AMF fungal sequences (House et al., 2016) using MAFFT (Katoh and Standley, 2013). This reference database consisted of sequences from the 350-bp D2 region of the nuclear-large subunit (LSU) rRNA gene in a previously published, Krüger et al. (2012) database with additional supplemental sequences from GenBank with confident species identification (House et al., 2016). We removed sequences that aligned poorly with the reference sequences and then created a rooted Maximum Likelihood (ML) phylogeny with the remaining consensus sequences and the reference sequences using RAxML (Stamatakis, 2014, RRID:SCR_006086), with *Mortierella elongata* as an outgroup. The rooted tree was used to remove any other OTU that did not cluster within the Glomeromycota database. All analyses were done for each site separately. Sequence counts were turned into proportions (total number of OTU sequences out of the total number of sequences in that sample) to account for variation in sequence number among samples.

To determine shared OTUs among sites, sequence data for each of the identified OTUs in all sites was compiled in alignment with the same in-house AMF database (House et al., 2016) to generate a maximum likelihood phylogenetic tree. Aligned sequences were uploaded to the CIPRES science gateway (Miller et al., 2010, RRID:SCR_008439) for analysis *via* RaxML (Stamatakis, 2014, RRID:SCR_006086)) using default settings with the following changes: 1,000 bootstrap iterations and print bootstrap values. Criteria for determining identical OTUs from the phylogenetic tree were as follows: (1) starting from the terminal node, collapse branches if support values are <70, otherwise retain original branching pattern, (2) cannot collapse branches where branching pattern is unresolved/polyphyletic, and (3) collapse split branches with ≥70 if there is no genetic distance between OTUs. Because one species of AMF can contain multiple OTUs (House et al., 2016), these collapsed OTUs often contained multiple OTUs from the same site. We call these collapsed OTUs “virtual OTUs” as they are similar in purpose to the virtual taxa designated by Opik et al. in the MaarjAM database (Opik et al., 2010).

### Other Metrics to Measure the Impact of Inoculation and Dispersal

#### Relative AMF Biomass

We also assessed how inocula dispersed and impacted the surrounding plant and soil community. Phospholipid fatty acid (PLFA) and neutral lipid fatty acid (NLFA) biomarker analyses were conducted to determine the relative abundances of extra-radical AMF. Phospholipid fatty acids are constituents of biological membranes that can be used to estimate the biomass of fungi (Tunlid and White, 1992), while neutral lipid fatty acids act as storage products and serve as the primary energy reserve in fungi (Larsen and Bødker, 2001). Total lipids were extracted from freeze-dried soil samples using a modification of the Bligh and Dyer (1959) extraction method described in detail by Allison and Miller (2005). The fatty acids were then analyzed by gas chromatography and mass spectrometry detection using Agilent GC 7890A/MS 5975C. Biomarkers 16:1ω5c, 22:1ω13 (tightly correlated with *Glomus* spp.), and 20:1ω9 (tightly correlated with *Gigaspora* spp.) were used to assess extra-radical AMF biomass.

#### Plant Community

We also assessed plant community identity along the nurse plant row, and 0.5 m, 1.0-, 1.5-, and 2.0-m away from the nurse plant row in the growing season between 2014 and 2016 using the point-intersect method (Middleton and Bever, 2012). The plant community was always assessed on the island side of the plot for Chanute, but at Tinker, the side (B or I) was randomly chosen each year.

#### Test Plants

At Chanute and Tinker, test plant species at different distances from the nurse plant row on both the island and bridge side were measured each growing season. Although these two sites contained different test plant species (*S. scoparium* at Chanute and *A. gerardii* at Tinker), we recorded leaf count and height at each site.

### Statistical Analysis

#### Dispersal of Inoculated OTUs

For each site, we first determined which AMF OTUs were present in the AMF and whole soil inocula used in each experiment. OTUs present in our inocula were often present in sites before inoculation or in sterile plots. Because of this, we could not use simple presence/absence at various distances and treatments to determine dispersal. We used separate Multivariate Analysis of Variance (MANOVA) tests to determine whether inoculated OTUs were more abundant in inoculated nurse plant rows compared to non-inoculated control nurse plant rows. We then used a separate MANOVA to determine whether inoculated OTUs decreased with distance from inoculated nurse plant rows, to determine the percentage of OTUs fitting into particular spread categories. Finally, for Chanute and Tinker, we used a separate MANOVA to determine whether inoculated OTUs were in greater abundance on the bridge compared to the island side at 2-m away. For all MANOVAs, we analyzed the site and year separately. All MANOVAs were performed in SAS (RRID:SCR_008567).

We categorized all present OTUs into the following spread categories, using contrasts of marginal means between each distance from the distance MANOVAs:

(1) No spread from the nurse plant row: OTU made up a significantly higher proportion of the AMF community in the nurse plant row compared to both 0.5- and 2-m away
(2) Spread 0.5-m away from the nurse plant row: OTU relative abundance in nurse plant row and 0.5-m away were not significantly different, but the OTU made up a significantly lower proportion of the AMF community 2-m away compared to other distances

We then have three categories involving varying levels of confidence in spread to 2-m away:

(3) Distance decay: the nurse plant row and 0.5-m away were not significantly different, 0.5-m and 2-m away were not significantly different, but the OTU made up a significantly higher proportion of the AMF community in the nurse plant row compared to 2-m away, or overall decrease with distance from the nurse plant row (although there was no statistically significant trend with distance, an overall distance effect was present, with the OTU decreasing in proportion to the rest of the community with distance)
(4) Spread to 2-m away: showed no significant differences across distance and inoculated nurse plant rows had higher relative abundance compared to non-inoculated nurse plant rows and
(5) Inconclusive distance effects (significant differences between different distances, but not displaying any clear distance effects or no differences across a distance or between inoculated and non-inoculated nurse plant rows).

At Chanute and Tinker, if there was a significantly higher proportion of the OTU on the bridge side compared to the island at 2-m away from the nurse plant row, and there was also no significant difference between the varying distances on the bridge side, we considered this spread to 2-m away. This information was assessed for Chanute in both years 1 and 2, and only the second year for Tinker. In the results, OTUs that spread to 2-m away include results from this analysis at these two sites.

We counted each OTU present in either the AMF or whole soil inocula as one trial. If an OTU was present in both, its spread category was assessed for both the AMF-inoculated plots and whole soil–inoculated plots. This resulted in 185 trials at Chanute (61 and 124 OTUs in the AMF and whole soil inocula, respectively), 133 trials for each Ft. Riley site (46 and 87 OTUs in the AMF and whole soil inocula, respectively), and 103 trials for Tinker (15 and 88 OTUs in the AMF and whole soil inocula, respectively). We used these trials to determine the proportion of OTUs in each spread category at each site and year.

#### Dispersal of OTUs Shared Among Sites

Using the virtual OTUs determined in the phylogenetic tree for all sites, we determined whether the same virtual OTU behaved similarly across sites. We assessed the proportion of trials for each virtual OTU that fit into one dominant spread category. As in other analyses, a trial is an OTU in a certain treatment, site, and year. We eliminated all trials that fit in the unknown spread category and then selected virtual OTUs that had at least 2 or more trials (19 total taxa) from at least two or more different original OTUs. We then examined whether these virtual OTUs had more than half of their trials in one particular spread category.

#### Dispersal of Glomeromycota Taxonomic Families

We tested for consistent differences in patterns of spread with different phylogenetic groupings using two approaches. First, using our entire dataset, we tested whether OTUs whose spread could be determined (i.e., spread categories 1–4), differed consistently between phylogenetic groupings. In practice, our power was limited by the few OTUs that could be confirmed to have spread and we, therefore, grouped all OTUs that could be determined to have spread out from their nurse plant (spread categories 2–4) and contrasted the likelihood of spreading to that of the absence of spread (spread category 0). Differences in the number of OTUs that spread between AMF families were tested using Chi-square tests in proc genmod in SAS (RRID:SCR_00856). This approach treated each OTU as an independent. Our second approach focused on the virtual OTUs that we were able to link between years, which allowed assessment of whether individual virtual OTUs spread across years. For this analysis, we used a generalized mixed model that tested the fixed effects of the AMF family and its interaction with Site and Year, and we identified OTU within the AMF family and interactions with year and site as a random effect. We used binomial error and logit links Proc Glimmix in SAS (RRID:SCR_00856).

#### Relative AMF Biomass

To assess the effects of inoculation on relative AMF abundances (through PLFA and NLFA analyses), generalized linear models (GLMs) were employed with the site, inoculum, distance from nurse plant row, and year as the main effects. Because of the left-skewed, positive nature of the data, GLMs with a gamma error distribution and log link were used.

To assess how AMF biomass varied between island and bridge sides for both Tinker and Chanute, we ran a separate generalized linear model for each site. In each model, we included treatment and side (island vs. bridge). Non-inoculated control plots were included at Tinker but not in the Chanute analysis. Both analyses used PLFA and NLFA data and were performed in base R (version 4.1.0).

#### Plant Community

Because plant community composition data were collected across the distance from the nurse plant row for all three treatments, we were able to analyze plant community richness, diversity, evenness, and composition across the distance for sterile control, whole soil, and AM fungi inocula plots in the one analysis. We used permutational multivariate analysis of variance (PERMANOVA) to assess how the plant community changed across sites, years, soil treatment, distance from nurse plant rows, and all interactions. We also included an experimental block within each site as a control. Because site and year were important in predicting plant community composition (see Results section), we also used PERMANOVA to assess plant community composition across inoculation treatment and distance from nurse plant row in specific sites and years. All PERMANOVAs were conducted using the adonis function in R.

We also used the vegan package in R to calculate richness, Shannon diversity, and evenness. Then, we used mixed effects models to assess how treatment, distance from nurse plant row, and the interaction impacted richness, Shannon diversity, and evenness across all sites and years. All mixed effect models had plot and block nested within site. We ran separate mixed effects models for specific sites and years to assess variation seen in the larger model. All mixed effect models were conducted using the PROC MIXED function in SAS.

#### Test Plants

We used a mixed effects model to assess how the test plant size (height and leaf number) at Chanute and Tinker varied by treatment, distance from the nurse plant row, and between the island and bridge side using the PROC MIXED function in SAS. Both leaf number and height were log transformed to meet assumptions of normality if necessary. We used a generalized linear mixed effects model to assess survival by the treatment, distance from nurse plant row, and between island and bridge in Chanute and Tinker using the PROC GLIMMIX function in SAS. Survival was estimated as a proportion of plants at each distance and bridge/island location for each plot, and then logit transformed to meet assumptions of normality. We conducted a separate analysis for each year for both plant size and survival.

## Results

### Molecular Analysis of Dispersal of Inoculated OTUs

#### Dispersal of Inoculated OTUs

**Figure 2:**
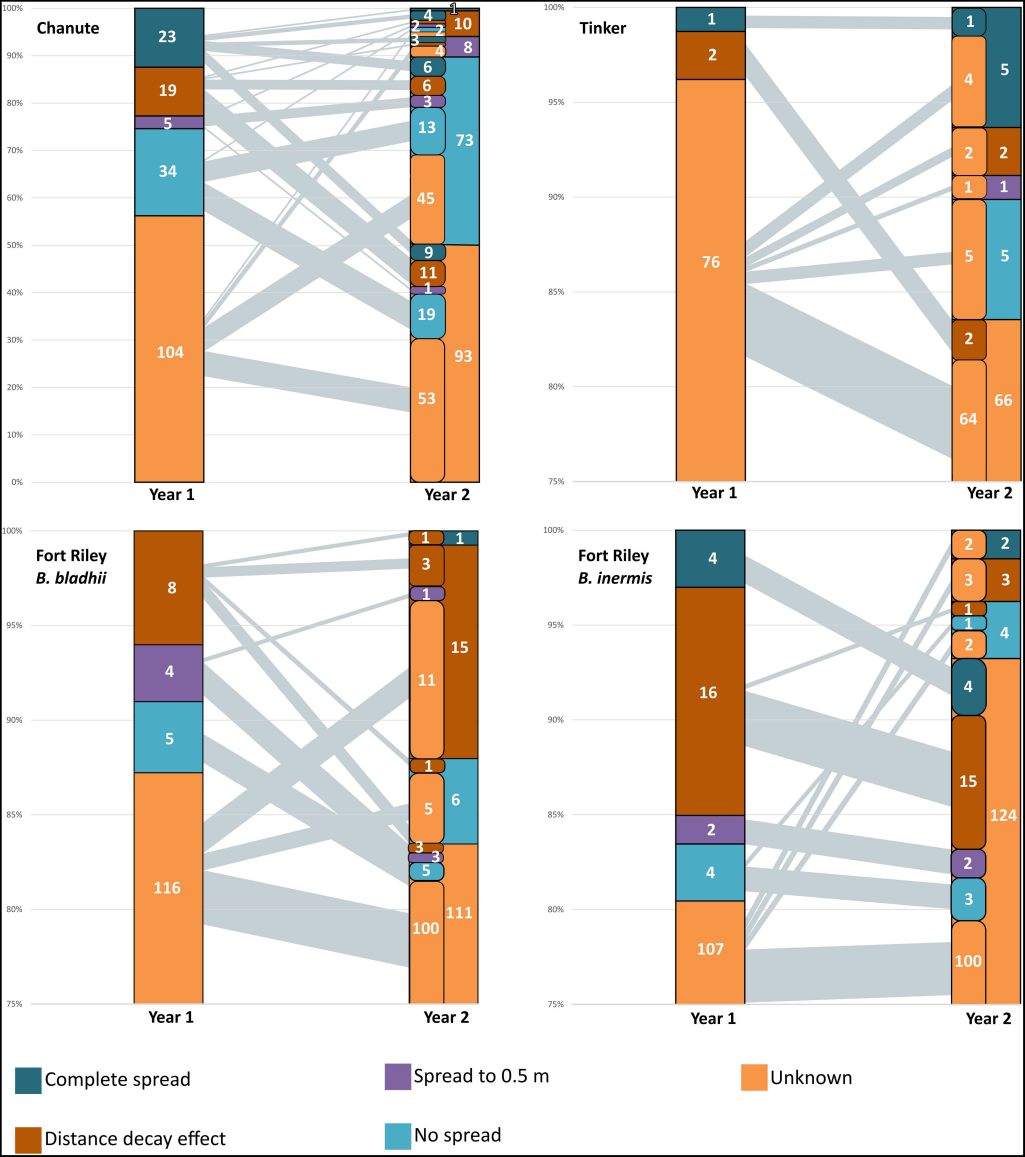
Spread of AMF OTUs from nurse plant row by sites in years 1 and 2. The number of OTUs in each spread category is presented. The left-side column in year 2 represents the contribution of OTUs from year 1 categories in each year 2 category. Black lines from year 1 to year 2 represent the amount of OTUs from categories in the first year to the second—the thicker the line, the greater the amount of OTUs from year 1 category in the year 2 category. The largest category in all sites was unknown, and there was evidence of the complete spread of some AMF in all sites by the second year.

The total number of observed AMF OTUs in our inocula treatments varied for each site: Chanute-185, Tinker-79, and Ft. Riley *B. inermis* and *B. bladhii*-133. The number of OTUs that were present in either one inocula treatment or that were common among both inocula treatments also varied between sites (Table 1). The ability to detect the presence of inocula OTUs and major spread patterns across inocula OTUs varied by site and year. For each site, OTUs that spread to either 0.5 or 2 m, failed to spread or were unable to be distinguished as having spread or not spread (unknown) in the first year often shifted into other spread categories in the second year (Figure 2). The largest category at every site was the unknown or undetermined spread category.

**Table 1:** The number of OTUs found in both AMF and Whole Soil inoculum treatment regimens at each site and the number of shared OTUs that showed different spread patterns in the two different treatments.

**Study site**	**Number of OTUs found in AMF and whole soil treatments**	**Number of OTUs in different spread categories in AMF vs. whole soil**
Ft. Riley *Bothriochloa bladhii*	42	16
Ft. Riley *Bromus inermis*	42	15
Tinker	11	2
Chanute	44	25

*For a detailed assessment of OTUs, see Supplementary Table S3*.

At Chanute in the first year, inocula OTUs overall made up a higher proportion of the AMF community in whole soil nurse plant rows compared to rows of non-inoculated nurse plants (F_1,16_ = 5.6, *p* < 0.05). Additionally, this proportion decreased precipitously with increasing distance from the nurse plant row for inoculated plots overall (${\text{F}}_{38}^{5}$ = 6.5, *p* < 0.001). Although the unknown spread category had the largest number of OTUs (104 out of 185 total, Figure 2), 34 OTUs did not significantly spread away from the nurse plant row, 5 spread to 0.5 m, 19 showed distance decay (declined with distance), and 23 OTUs were confirmed to spread at least 2-m away from the nurse plant row (Figure 2). In the second year, inoculated nurse plant rows had a marginally significantly greater abundance of inoculated OTUs compared to non-inoculated nurse plant rows (${\text{F}}_{16}^{1}$ = 3.9, *p* < 0.1). More specifically, the AMF-only inoculation plots had a greater proportion of inocula OTUs compared to non-inoculated plots (${\text{F}}_{16}^{1}$ = 4.5, *p* = 0.05). The overall distance effect remained in the second year, with an abundance of inoculated OTUs decreasing with distance from the nurse plant row (${\text{F}}_{37}^{5}$ = 30.8, *p* < 0.0001). The unknown category was again the largest (93 OTUs out of 185 total), but 73 OTUs did not spread away from the nurse plant row, with 13 of those remaining in that category from the following year. Two OTUs spread further in the second year, while 22 OTUs moved backward *via* the spread category, being detected closer to the nurse plant row in the second year compared to the first year (Figure 2).

At Ft. Riley *B. inermis*, Ft. Riley *B. bladhii*, and Tinker, inoculated OTUs were not more abundant in inoculated nurse plant rows compared to non-inoculated nurse plant rows, although at Tinker they were marginally significantly more abundant in whole soil compared to non-inoculated control nurse plant rows in the second year (${\text{F}}_{52}^{1}$ = 3.1, *p* = 0.08). At Tinker in the second year, inocula OTUs did vary marginally across distance (${\text{F}}_{30}^{5}$ = 2.48, *p* = 0.054). This was driven by differences between the nurse plant row and 0.5 m distance in whole soil plots (${\text{F}}_{30}^{1}$ = 4.79, *p* < 0.05) and, overall, differences in inoculated plots between the nurse plant row and 0.5 m (${\text{F}}_{30}^{1}$ = 3.2, *p* < 0.1) and 0.5-m and 2.0-m (${\text{F}}_{30}^{1}$ = 3.8, *p* < 0.1).

In all three of these sites, just as in Chanute, OTUs were in different spread categories for each site and year (Figure 2), but OTUs with an unknown or undetectable spread pattern made up 80–95% of the OTUs in both years. For Ft. Riley *B. bladhii*, Ft. Riley *B inermis*, and Tinker, 89, 92, and 100%, respectively, of the OTUs in a detectable spread category in the first year were in the unknown category in the second year (Figure 2).

At each study site, some proportion of OTUs were found in both the whole soil and the AMF inoculum treatments. These OTUs were recorded and often showed different spread characteristics in whole vs. AMF-inoculated plots (Supplementary Table S3). Chanute had the largest proportion of OTUs in both whole and AMF inocula in different spread categories (25 of 44).

At Chanute, there was a marginally significantly higher proportion of inoculated OTUs at the Bridge side 2-m away from the nurse plant row compared to the island side (${\text{F}}_{21}^{1}$ = 4.1, *p* = 0.06) in the first year, but no significant differences overall in the second year. At Tinker, there was not a significant difference in the abundance of the inocula OTUs between the island and bridge overall.

#### Dispersal of Glomeromycota Taxonomic Families

**Figure 3:**
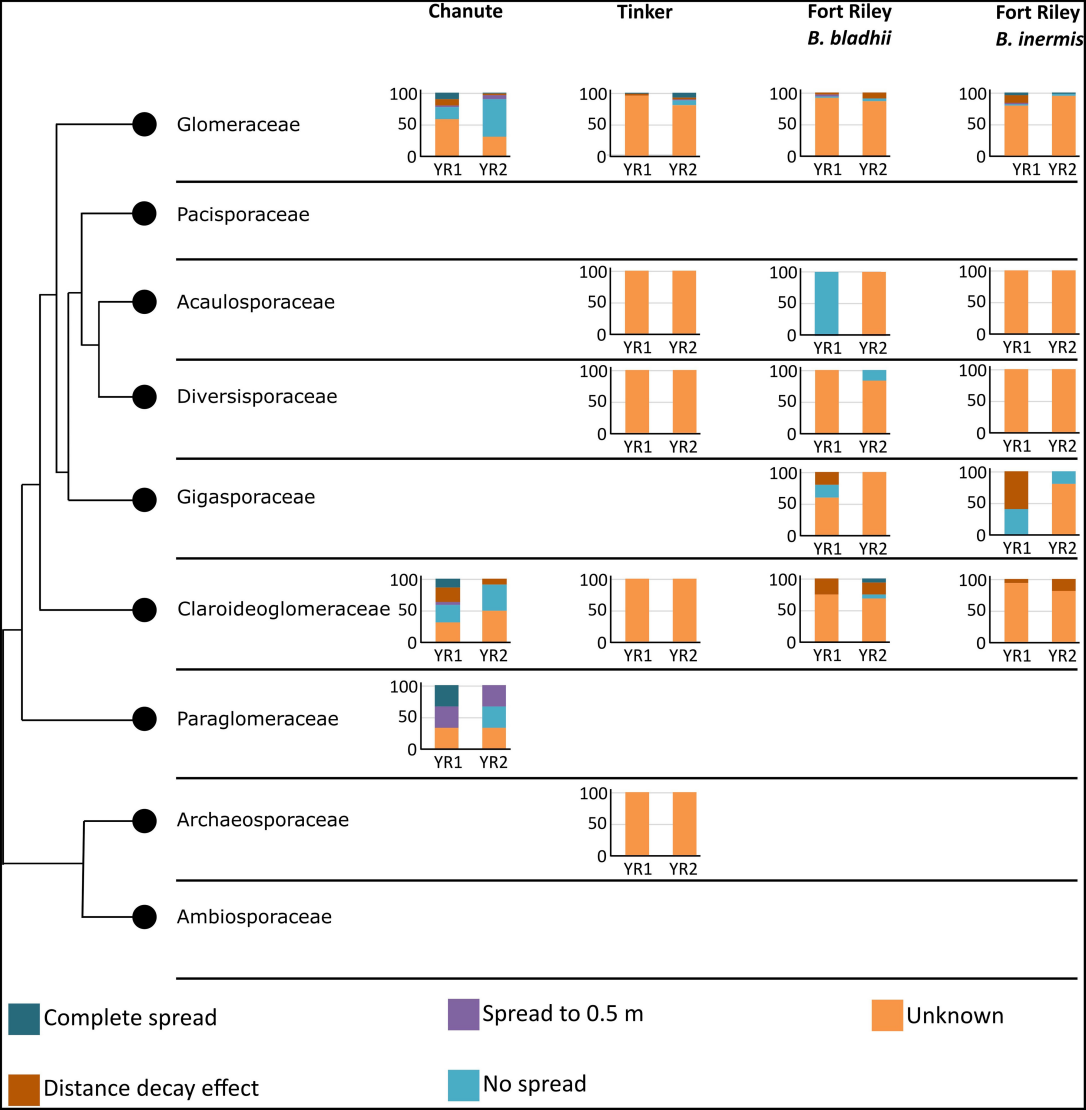
Spread categories by taxonomy. Y-axis represents the percentage of trials in each category. Graphs are broken down into site and year. Colors represent the spread categories.

**Figure 4:**
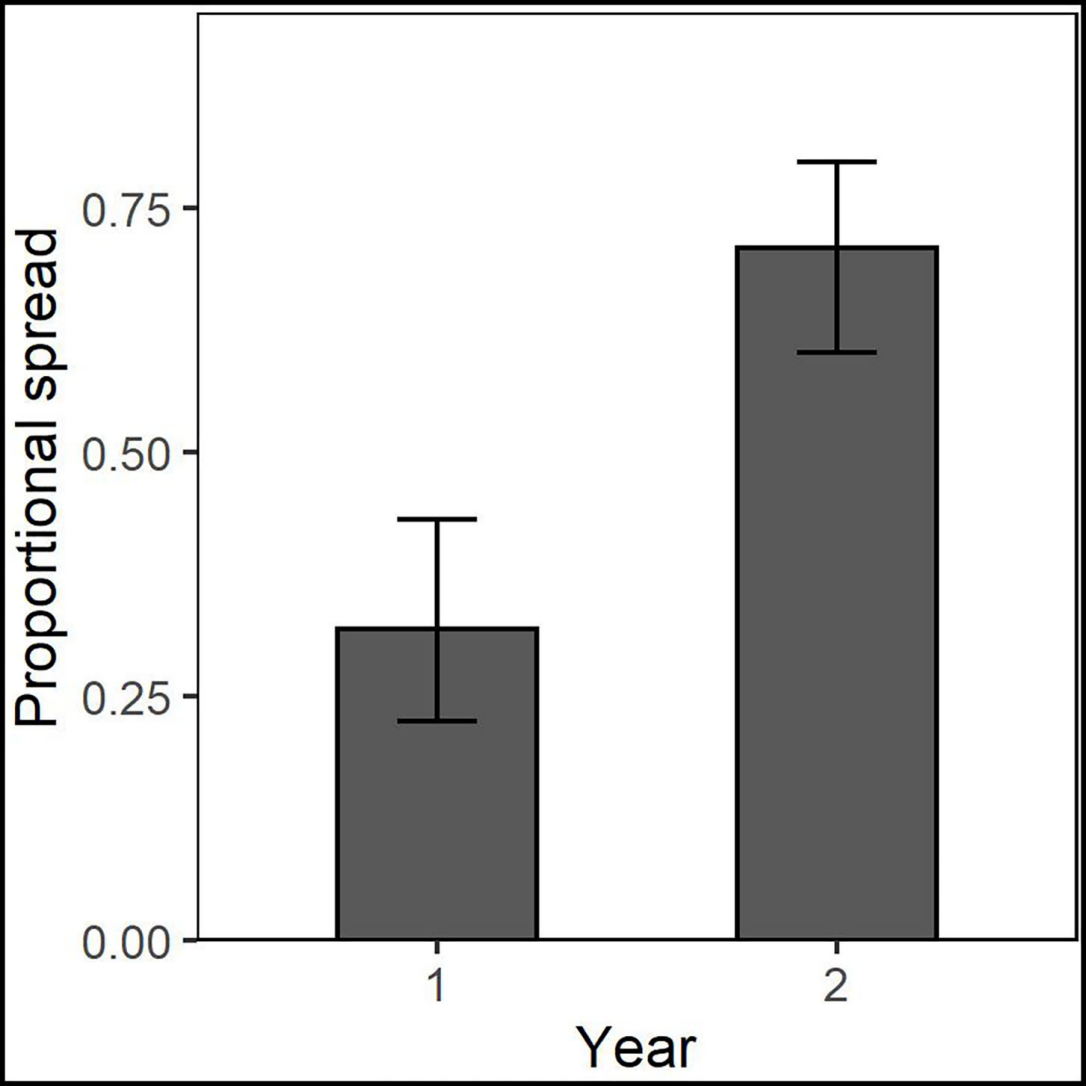
Common OTUs identified across years within the Chanute site were more likely to spread from the nurse plant row in the second year.

Taxonomic placement of OTUs found in the different spread categories resulted in 105 OTUs identified to family in Chanute, 74 in Tinker, and 125 in Ft. Riley. These data were then used to determine spread patterns at each site in each AMF family (Figure 3). The greatest number of OTUs to spread through the soil in the three spread categories occurred in the Glomeraceae and Claroideoglomeraceae families at Chanute (18 and 9, respectively, year 1), and the Glomeraceae at the Ft. Riley *B. inermis* site (18 in year 1). OTUs assigned to families were used in a statistical analysis of the spread by the family across the sites each year. No strong relationship was found between any family and their ability to spread in the soil substrate. At Chanute, which had the most data, Paraglomerales tended to be more likely to spread from the nurse plant row than Glomerales (χ^2^ = 3.48, *p* = 0.06). Common OTUs identified across years within the Chanute site were more likely to spread from the nurse plant row in the second year (${\text{F}}_{35}^{1}$ = 12.38, *p* = 0.001, Figure 4; Supplementary Table S4) and Glomeraceae tended to be more likely to spread from the nurse plant row than Claroideoglomeraceae (${\text{F}}_{35}^{1}$ = 3.09, *p* = 0.088). PLFA biomarkers indicated no significant shift in biomass of genera *Glomus* and *Gigaspora*.

#### Spread of OTUs Shared Among Sites

Of those 15 OTUs containing two or more trials across more than one site, 13 of them showed more than 50% of their trials in one specific category, which is ~87% of those OTUs shared among sites (Table 2). Of these, 11 OTUs were dominated by the no spread category, one was dominated by the spread to 0.5 category, and 2 were dominated by the distance decay category.

**Table 2:** Trials of virtual OTUs across sites.

**Virtual OTUs**	**Number of trials**	**Number of non-unknown trials**	**Sites with non-unknown trials**	**No spread**	**Spread to 0.5-m**	**Distance decay effect**	**Complete spread to 2-m**
1	10	2	Chanute, Ft. Riley *B. bladhii*	100%	0	0	0
2	20	7	Chanute, Ft. Riley *B. bladhii*, Ft. Riley *B. inermis*	86%	0	14%	0
3	42	18	Chanute, Ft. Riley *B. inermis*, Tinkr	78%	0	11%	11%
4	46	17	Chanute, Ft. Riley *B. bladhii*, Tinkr	53%	18%	29%	0%
7	12	2	Ft. Riley *B. bladhii*, Tinker	100%	0	0%	0
9	12	3	Chantue, Ft. Riley *B. inermis*	33%	0	0%	67%
10	6	2	Chanute	100%	0	0%	0%
14	14	4	Chanute	75%	0	25%	0%
16	14	2	Chanute	0%	0	50%	50%
17	12	4	Chanute	100%	0	0%	0%
18	12	4	Chanute	100%	0	0%	0%
19	6	2	Chanute, Ft. Riley *B. inermis*	0%	100%	0%	0%
20	10	3	Chanute, Ft. Riley *B. inermis*	67%	0	33%	0%
21	6	4	Chanute	75%	0	25%	0%
22	6	2	Tinker	0%	0	50%	50%

*This table only includes the virtual OTUs that had more than one trial (one trial= an OTU in treatment in a year) when all trials that fit into an unknown spread category were removed. Once the trials that fit into an unknown category were removed, some virtual OTUs only had trials on one site*.

### Other Metrics to Measure the Impact of Inoculation and Dispersal

#### Relative AMF Biomass

**Figure 5:**
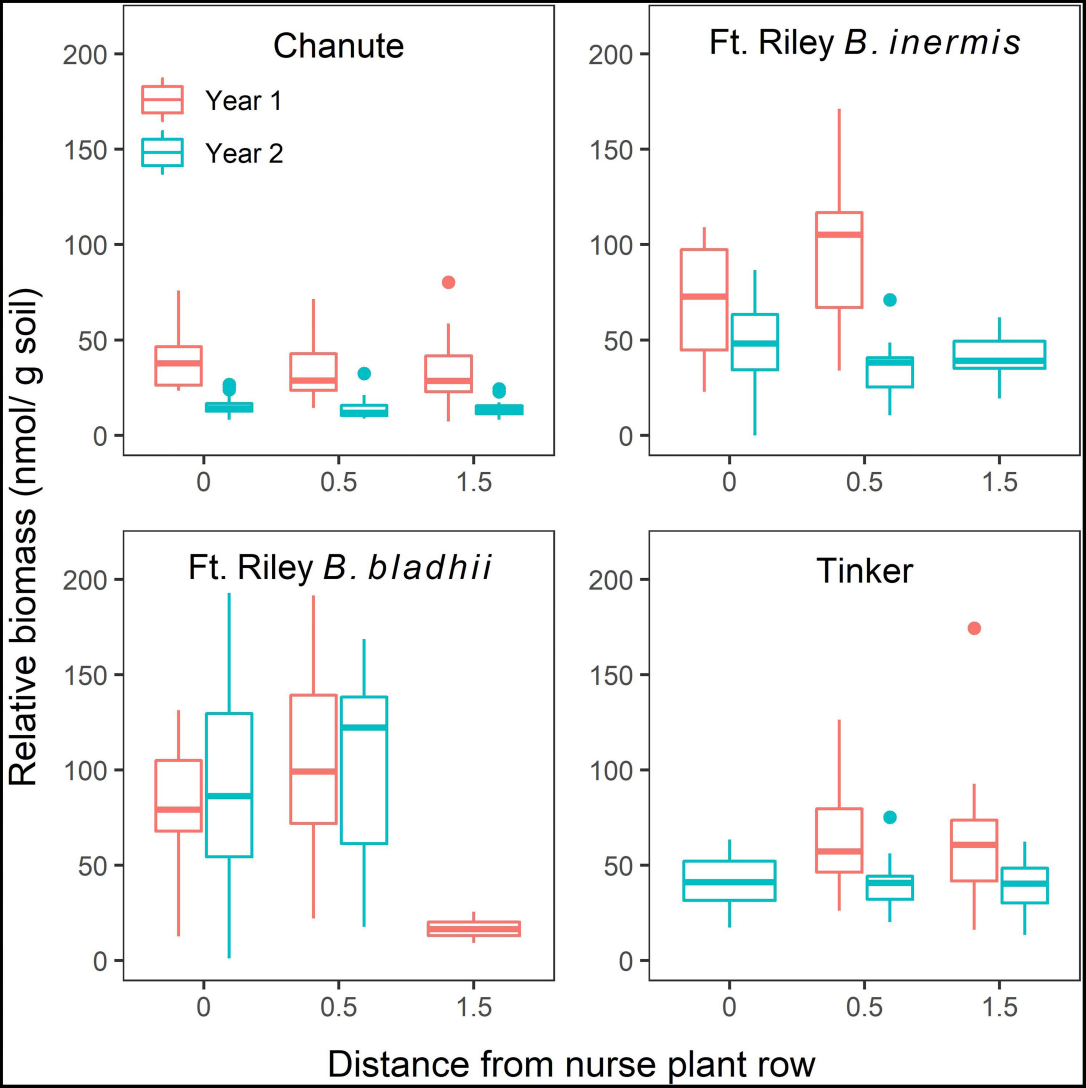
The effects of site, distance from nurse plant row (X-axis), and year on relative abundances of extra-radical arbuscular mycorrhizal (AM) fungi (mean ± SE) measured through NLFA. Red bars represent measurements from year 1, and blue bars represent measurements from year 2.

A significant three-way interaction between site, distance from nurse plant row, and the year was detected when utilizing both NLFA (${\text{F}}_{61}^{5}$ = 2.80, *p* < 0.03; Supplementary Table S5) and PLFA (${\text{F}}_{54}^{5}$ = 38.5, *p* < 0.0001; Supplementary Table S6), with general decreases from year 1 to 2, as well as with increasing distances from the nurse plant row (Figure 5; Supplementary Figure S1).

When comparing the bridge vs. the island side of plots at 1.5 m from the nurse plant row at Tinker through NLFA, no significant effects of side or treatment, or the interaction, were detected (Supplementary Figure S2). However, through PLFA analyses, there was a significant side to treatment interaction (${\text{F}}_{54}^{2}$ = 3.1, *p* < 0.05). In both the whole soil and sterile control plots, AMF biomass at 1.5-m away was higher on the island side compared to the bridge side. But, in the AMF plots, the bridge side had slightly higher AMF biomass than the island side (Supplementary Figure S3). At Chanute, there was a significant effect of side, with significantly greater relative AMF biomass on island sides (${\text{F}}_{54}^{2}$ = 6.68, *p* = 0.01), and this difference was more noticeable in the AMF treatment, relative to whole soil (Supplementary Figure S4). There was no significant difference in relative AMF biomass measured through PLFAs between the island and bridge side at Chanute (Supplementary Figure S5).

#### Plant Community

**Figure 6:**
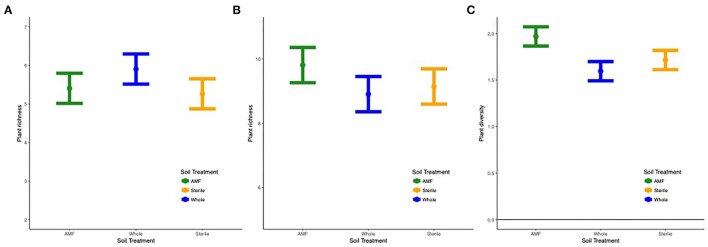
**(A)** Plant richness across the three soil treatments in **(A)** Tinker site across all years and **(B)** Ft. Riley *B. bladhii* site in year 1 and **(C)** diversity in Ft. Riley *B. inermis* site in year 3.

Overall, richness (${\text{F}}_{78}^{3}$ = 9.7, *p* < 0.0001), diversity (${\text{F}}_{78}^{3}$ = 4.3, *p* < 0.01), and plant community composition (NMDS1, ${\text{F}}_{78}^{3}$ = 18.1, *p* < 0.0001; NMDS2, ${\text{F}}_{78}^{3}$ = 4.4, *p* < 0.01) varied by site and year. In all sites except Ft. Riley *B. bladhii*, richness and diversity increased across time (Supplementary Figure S6). Within specific sites and years results varied, but when soil treatment was a significant predictor of plant diversity, richness, or evenness, inoculated plots (AMF and whole soil) or AMF plots alone had significantly higher plant diversity values. Diversity (${\text{F}}_{52}^{6}$ = 2.3, *p* < 0.05) and evenness (${\text{F}}_{78}^{6}$ = 2.7, *p* = 0.02) also differed by soil treatment, year, and site, which was driven by higher diversity and evenness in AMF compared to whole plots in the third year at Ft. Riley *B. inermis* (Figure 6; Supplementary Figures S7, S8).

Both sites with monocultures of *B. bladhii* before restoration efforts (Ft. Riley *B. bladhii* and Tinker) displayed significant differences in plant richness, diversity, and evenness across soil treatment and distance in certain years. At Tinker, inoculated plots (AMF and whole plots) had a marginally significantly higher plant richness than sterile plots overall (${\text{F}}_{12}^{1}$ = 3.2, *p* < 0.1, Figure 6A). At *Ft. Riley B. bladhii*, plant richness was significantly higher in AMF plots compared to both sterile and whole plots in the first year (${\text{F}}_{12}^{2}$ = 4.5, *p* < 0.05; Figure 6B), although there were no significant differences in the second or third year. Both these sites also showed some significant changes with distance from the nurse plant row. At Ft. Riley *B. bladhii*, plant richness in year 1 and plant evenness in year 2 decreased away from the nurse plant row in AMF plots but increased in whole soil plots with distance from the nurse plant row (${\text{F}}_{39}^{2}$ = 4.1, *p* < 0.05; ${\text{F}}_{39}^{1}$ = 3.8, *p* < 0.1; Supplementary Figures S9A,B). In the third year, both AMF and whole soil plots had the highest plant richness (${\text{F}}_{39}^{1}$ = 4.3, *p* < 0.05) and diversity (${\text{F}}_{39}^{1}$ = 4.4, *p* < 0.05) near the nurse plant row compared to sterile plots, where richness and diversity increased with distance (Supplementary Figures S9C,D). At Tinker overall, plant community evenness decreased with distance in AMF plots and increased in whole plots (${\text{F}}_{96}^{1}$ = 4.1, *p* < 0.05, Supplementary Figure S9E).

Plant community composition (PERMANOVA) varied by soil treatment at both sites originally dominated by *B. bladhii*. At Tinker, the plant community was significantly different across the soil treatments, regardless of distance from the nurse plant row (${\text{F}}_{112}^{2}$ = 2.4, *p* < 0.01, Supplementary Figure S10C). At the Ft. Riley *B. bladhii* site, plant community composition was significantly different across soil treatments in the first year (${\text{F}}_{55}^{2}$ = 1.9, *p* < 0.05, Supplementary Figure S10A), and marginally significantly different across inoculation treatment in the third year (${\text{F}}_{55}^{2}$ = 1.9, *p* < 0.1, Supplementary Figure S10B). Soil treatment was also important for explaining community composition in the third year at Ft. Riley *B. inermis* (${\text{F}}_{55}^{2}$ = 2.0, *p* < 0.05, Supplementary Figure S10D).

#### Test Plants

At Tinker, test plants in inoculated plots were larger overall (${\text{F}}_{12}^{2}=$ 5, *p* < 0.05, Supplementary Figure S11). There was also a significant distance effect (${\text{F}}_{66}^{4}$ = 7, *p* < 0.0001), where test plant species in the nurse plant row were larger compared to all other distances (Supplementary Figure S11), but the bridge and island sides did not vary significantly in size. Although the size of the plants varied by treatment and distance at Tinker, survival did not vary across any experimental variables. Survival of *S. scoparium* test plants was high across all distances, treatments, and years in Chanute, with 90% of the plants surviving to the third year. Test plant size and survival at Chanute varied with distance and side (island vs. bridge) in year 3 but did not follow any pattern moving toward or away from the nurse plant row.

## Discussion

This work evaluates AMF spread across four experiments distributed across three states and indicates that rates of AMF spread vary among taxa, are context dependent, and can impact neighboring plant communities. We found that many members of the AMF community were generally slow to spread from the site of inoculation, with most detectable OTUs (those not in the unknown category) either not spreading, spreading only to the 0.5 m, or spreading with a distance decay function. These results are supported by our findings that increases in relative AMF biomass were also slow, with increasing distances from the sites of inoculation. Together, these results indicate that AMF is both slow to spread and slow to increase in abundance from the site of colonization or inoculation—but that they can indeed spread. This result is consistent with previous studies showing variation in the mycorrhizal community closer to and away from mycorrhizal hosts (Chaudhary et al., 2014) and with evidence of the benefits of inoculation of native AMF spreading several meters from points of inoculation (Middleton and Bever, 2012; Middleton et al., 2015). We found the rate of the dispersal of AMF in the soil varies strongly among taxa, but that spread was not strongly predicted by the family. Generally, the proportion of AMF dispersing to 2 m is dependent on the presence of high-quality plant hosts and is affected by environmental context.

Environmental variation between sites likely influenced AMF spread from inoculation points, and thus our ability to detect this spread. For example, at Chanute, the AMF community was greatly reduced at a broad spatial scale through whole site disking and removal of existing vegetation. Because of these management procedures, we were able to better detect spread patterns for a high proportion of inoculated OTUs. However, at Tinker and Ft. Riley sites, where vegetation in areas outside of the plot tarp treatment was not removed, competition from other AMF or dispersal of AMF OTUs from outside the plot may explain the large abundance of OTUs displaying non-significant or unknown spread patterns in these sites. In support of this dispersal from the edge hypothesis, plots at Ft. Riley *B. inermis* generally showed an increase in AMF biomass moving away from the nurse plant row (calculated *via* PLFA) patterns in the second year of the experiment (Supplementary Figure S1). Sites also varied in dominant plant species before restoration. Previous studies have shown that land use history, soil nutrient levels, and dominant plant species can alter AMF communities (House and Bever, 2018; Tipton et al., 2018) and that mycorrhizal communities vary most at the site or regional scale (Chaudhary et al., 2014). This study suggests that site history may also alter either the spread of AMF from inoculation sites or the ability to detect this spread using molecular techniques in the field.

The use of island and bridge host plants on either side of the inoculation row allowed us to further distinguish spread rates among plant hosts, where hosts on the bridge side aided the spread of native inoculated AMF away from the inoculated nurse plants. Other studies have shown the benefits of the spread of inoculated native AMF to prairie plant growth up to 2 m in distance (Middleton and Bever, 2012; Middleton et al., 2015). Our results suggest that the rates of spread and benefit will depend in part on the establishment of mycotrophic host plants. In the prairie system, late-successional plant species are more responsive to native inocula than early-successional native and non-native plant species that establish quickly from seed and dominate the first several years of restoration (Cheeke et al., 2019). We showed that planting a row of non-inoculated late-successional host plant species at 0.5 m distances (the bridge) resulted in a greater proportion of inoculated OTUs spreading to 2.0 m and greater PLFA abundances compared to weedy host plants that dominated from seed (the island). Although we did not see significant differences in survival of these test plants with distance from inoculated nurse plant rows, *A. gerardii* plants were larger in the nurse plant row compared to all other distances (Supplementary Figure S11), suggesting that inoculation did impact overall growth. We also know both *S. scoparium* and *A. gerardii* are hosts of native AMF (Anderson et al., 1994) and could facilitate AMF hypha through the soil. Chaudhary et al. (2014) found that spore abundance but not hyphal density varied between shrub understory and un-vegetated open space 1-m away, suggesting that hyphae may be able to spread at least 1 m from a host. However, these were established shrubs and the taxonomic identity of these hyphae was not tested. It could be that some AMF taxa rely more on the presence of a plant host for dispersal, compared to other taxa. In restoration, when new plant vegetation is added, presence of a host may be essential for dispersal of some AMF taxa. Although we did not examine how bridge and island sides varied in plant community diversity, future studies should examine how existing host plants could improve the spread of AMF inocula and thus increase abundance of late successional and AMF-dependent plant species away from inoculation points.

Our assessment of dispersal patterns as a function of AMF taxonomy yielded mixed results. Our data showed only weak patterns of OTUs in Paraglomerales tending to spread more than those in Glomerales and OTUs in Glomeraceae tending to spread more than those in Claroideoglomeraceae within the Chanute restoration site. However, when assessing the spread of the same OTUs at different sites, there appeared to be some support for identical spread patterns across varied environmental, host, and land use history parameters (Supplementary Table S3). Moreover, at the Chanute site, individual OTUs likelihood of spread increased from year 1 to year 2. Therefore, these data suggest that the spread of AMF through soil by hyphal growth is not solely dictated by the environment, but varies depending on the fungal taxon in question. This is in line with previous research on hyphal growth in soil for other fungal groups (Friese and Allen, 1991).

Given that so many native plant species are dependent on mycorrhizae (Koziol and Bever, 2015; Bauer et al., 2018; Cheeke et al., 2019) and that the presence of specific species of mycorrhizal fungi can strongly influence the growth of native plant species (Koziol and Bever, 2016; Cheeke et al., 2019), the rate of AMF spread from sites of colonization could have important impacts on the resulting plant community that occurs as AMF spread or does not spread. Our results support that slow AMF spread from inoculation sites limited plant community establishment. For instance, as we observed fungal OTUs spreading across distance and years, we observed increases in plant community richness and diversity across years with amendment with AMF or whole soil inoculum at some sites (Supplementary Figures S4, S5; Figure 6). Although whether or not these inocula had a significant impact on the plant community differed between year and site, when there was a significant effect, it included an increase in diversity and richness in AMF-inoculated plots (Supplementary Figures S7, S8; Figure 6), suggesting these soil amendments have the potential to alter the plant community at scales beyond the nurse plant level. Past work has also indicated that the presence of specific fungi in the field can increase seed recruitment, diversity, and native richness (Koziol and Bever, 2017) and that these benefits of AMF increase with the density of native AMF inocula (Koziol et al., 2020).

This work expands much-needed work on dispersal mechanisms of AMF, especially how dispersal *via* soil varies among taxa and environmental contexts. However, determining the dispersal of many AMF taxa was difficult using this approach in the field. Many of the AMF OTUs present in our inocula were also present at a background level in sites before the experiment, leaving us unable to determine dispersal patterns for some taxa. Different tools should be utilized in the future to expand our knowledge of dispersal, including controlled experiments involving multiple taxonomic groups, to determine the propagule type and dispersal rate of multiple species. A better understanding of AMF dispersal can lead to more efficient plans for the reintroduction of these fungi, to the benefit of the plant species that depend on them, and to the improvement of ecological restoration.

## Data Availability Statement

The data presented in the study are deposited in the NCBI repository, accession number PRJNA818716.

## Author Contributions

JB, PS, and GW conceived and designed the experiment. AT, GH, ED, and LK collected data. AT, DN, GH, LK, and JB analyzed the data. AT wrote the manuscript. All authors contributed toward the materials and edits to manuscript drafts. All authors contributed to the article and approved the submitted version.

## Funding

This work was supported by U.S. Department of Defense, Grant/Award Number: SERDP (RC-2330) and National Science Foundation, Award Numbers: DEB 1556664, PFI 2106549, and OIA 1656006.

## Conflict of Interest

GH was employed by NEON. The remaining authors declare that the research was conducted in the absence of any commercial or financial relationships that could be construed as a potential conflict of interest.

## Publisher's Note

All claims expressed in this article are solely those of the authors and do not necessarily represent those of their affiliated organizations, or those of the publisher, the editors and the reviewers. Any product that may be evaluated in this article, or claim that may be made by its manufacturer, is not guaranteed or endorsed by the publisher.
