# Supplementary material for: Arbuscular Mycorrhizal Fungi Taxa Show Variable Patterns of Micro-Scale Dispersal in Prairie Restorations
Source: Front Microbiol. 2022 Jul 22;13:827293. doi: 10.3389/fmicb.2022.827293 (PMC9355535; doi:10.3389/fmicb.2022.827293)

Supplementary Information

Text S1
Methods for creation of AMF inocula

To create single spore inocula from each site, we filled pots with autoclaved 1:1 sand and soil mixture, then inoculated pots with spores from a single AMF isolate. *Sorghum bicolor* was planted in each pot and used as the trap plant for all cultures. After a six to nine month growing season, spore-cultures were harvested by removing aboveground biomass and storing sand: soil mixture and *S. bicolor* root biomass at 4°C. We again extracted spores from each pot to confirm spore production and identity, and then homogenized soil and roots to inoculate new cultures the following growing season. This process was conducted twice before AMF inocula were applied to nurse plants.

Text S2
Methods for nurse plant growth and inoculation

Nurse plant species (Table S1) were cold moist stratified for 30-60 days before germination in autoclaved sand in the spring and were planted into inocula treatments within 20 days following germination. Seedlings were inoculated with either whole soil inocula, AM fungal inocula, or sterilized inocula from background soil. Henceforth we refer to inoculated plants as "nurse plants." Nurse plants were inoculated with microbial treatments at 15% by volume (23 cm^2) at the center depth of 150 cm^3 conetainers™ with the remaining volume containing autoclaved soil:sand mixtures. Soils used in the nurse plant pots were collected from each restoration site and sterilized via autoclaving for two hours (see Supplementary Information 2 for soil nutrient analysis). We selected mid-late successional plants that are frequently seeded into restoration to use as nurse plants. We included two forbs, one grass, and one legume at each restoration site (Table S1) and included *Allium cernuum, Amorpha canescens, Schizachyrium scoparium, Echinacea pallida, Echinacea angustifolia, Allium stellatum, Andropogon gerardii, Lespedeza capitate, Dalea purpurae, Ratibida pinnata.* Nurse plants for the Oklahoma and Kansas restorations were grown in the Oklahoma State University greenhouses in Stillwater, OK and nurse plants for the Illinois restoration were grown under similar conditions in the Indiana University greenhouses in Bloomington, IN under ambient light during April of 2014 or 2015. Inoculated seedlings were grown for 21-25 days in the greenhouse prior to being transplanted in the field in the spring. Following transplant, seedlings were watered twice at each site, once at planting and once two weeks after planting, to facilitate first year establishment. Once plants were established, we no long watered plants. Initial plant size (height and/or leaf or tiller number) was measured at time of transplanting into plots at each restoration site, and again annually. At the Chanute site, grass height was not collected for the second growing season, and grass tiller number was not collected the third growing season. We report plant growth and survival at the end of each growing season.

Supplementary Tables and Figures

Table S1. List of site locations, year planted with nurse plants, nurse plants species used, number of blocks, and pre-restoration site preparation.

Site	Site Abreviation	Prior Dominant Vegetation	Location	Year Planted	Nurse Plant Speceis Used	Blocks	Was existing vegetation removed?	Pre-restoraiton site preparation
Chanute Airforce Base	Chanute	*Schedonorus arundinaceus*	Rantoul, IL	2014	*Allium cernuum*	9	Yes	Sprayed with __% Glyphstae, Disked
Chanute Airforce Base	Chanute	*Schedonorus arundinaceus*	Rantoul, IL	2014	*Amorpha canescens*	9	Yes	Sprayed with __% Glyphstae, Disked
Chanute Airforce Base	Chanute	*Schedonorus arundinaceus*	Rantoul, IL	2014	*Schizachyrium scoparium*	9	Yes	Sprayed with __% Glyphstae, Disked
Chanute Airforce Base	Chanute	*Schedonorus arundinaceus*	Rantoul, IL	2014	*Echinacea pallida*	9	Yes	Sprayed with __% Glyphstae, Disked
Ft. Riley Army Base	Ft. Riley *B. inermis*	*Bromus inermis*	Junction City, KS	2014	*Echinacea angustifolia*	7	Yes	Tarped for 4 weeks
Ft. Riley Army Base	Ft. Riley *B. inermis*	*Bromus inermis*	Junction City, KS	2014	*Allium stellatum*	7	Yes	Tarped for 4 weeks
Ft. Riley Army Base	Ft. Riley *B. inermis*	*Bromus inermis*	Junction City, KS	2014	*Andropogon gerardii*	7	Yes	Tarped for 4 weeks
Ft. Riley Army Base	Ft. Riley *B. inermis*	*Bromus inermis*	Junction City, KS	2014	*Amorpha canescens*	7	Yes	Tarped for 4 weeks
Ft. Riley Army Base	Ft. Riley *B. inermis*	*Bromus inermis*	Junction City, KS	2015	*Lespedeza capitata*	7	No	None
Ft. Riley Army Base	Ft. Riley *B. inermis*	*Bromus inermis*	Junction City, KS	2015	*Dalea purpurae*	7	No	None
Ft. Riley Army Base	Ft. Riley *B. inermis*	*Bromus inermis*	Junction City, KS	2015	*Ratibida pinata*	7	No	None
Ft. Riley Army Base	Ft. Riley *B. inermis*	*Bromus inermis*	Junction City, KS	2015	*Andropogon gerardii*	7	No	None
Ft. Riley Army Base	Ft. Riley *B. bladhii*	*Bothriochloa bladhii*	Junction City, KS	2014	*Echinacea angustifolia*	7	Yes	Tarped for 4 weeks
Ft. Riley Army Base	Ft. Riley *B. bladhii*	*Bothriochloa bladhii*	Junction City, KS	2014	*Allium stellatum*	7	Yes	Tarped for 4 weeks
Ft. Riley Army Base	Ft. Riley *B. bladhii*	*Bothriochloa bladhii*	Junction City, KS	2014	*Andropogon gerardii*	7	Yes	Tarped for 4 weeks
Ft. Riley Army Base	Ft. Riley *B. bladhii*	*Bothriochloa bladhii*	Junction City, KS	2014	*Amorpha canescens*	7	Yes	Tarped for 4 weeks
Ft. Riley Army Base	Ft. Riley *B. bladhii*	*Bothriochloa bladhii*	Junction City, KS	2015	*Lespedeza capitata*	7	No	None
Ft. Riley Army Base	Ft. Riley *B. bladhii*	*Bothriochloa bladhii*	Junction City, KS	2015	*Dalea purpurae*	7	No	None
Ft. Riley Army Base	Ft. Riley *B. bladhii*	*Bothriochloa bladhii*	Junction City, KS	2015	*Ratibida pinata*	7	No	None
Ft. Riley Army Base	Ft. Riley *B. bladhii*	*Bothriochloa bladhii*	Junction City, KS	2015	*Andropogon gerardii*	7	No	None
Tinker Airforce Base	Tinker	*Bothriochloa ischaemum*	Oklahoma City, OK	2015	*Lespedeza capitata*	7	Yes	Tarped for 60 weeks
Tinker Airforce Base	Tinker	*Bothriochloa ischaemum*	Oklahoma City, OK	2015	*Dalea purpurae*	7	Yes	Tarped for 60 weeks
Tinker Airforce Base	Tinker	*Bothriochloa ischaemum*	Oklahoma City, OK	2015	*Ratibida pinata*	7	Yes	Tarped for 60 weeks
Tinker Airforce Base	Tinker	*Bothriochloa ischaemum*	Oklahoma City, OK	2015	*Andropogon gerardii*	7	Yes	Tarped for 60 weeks

Table S2. Distances from the nurse plant row that were assessed for AMF biomass at each site for each year.

Site	Year of Experiment	Distance Assessed (m)
Chanute	1	0, 0.5, and 1.5
Chanute	2	0, 0.5, and 1.5
Ft. Riley *B. inermis*	1	0, 0.5
Ft. Riley *B. inermis*	2	0, 0.5, 1.5
Ft. Riley *B. bladhii*	1	0, 0.5, 1.5
Ft. Riley *B. bladhii*	2	0, 0.5, 1.5
Tinker	1	0, 0.5, 1.5
Tinker	2	0, 0.5, 1.5

Table S3. Detailed list of OTUs shared between AMF and Whole soil inoculum treatments at 4 study sites, and the spread categories for each OTU.

Study Site	OTU	Trial	Category Year 1	Category Year 2	Trial	Category Year 1	Category Year 2
Tinker	9	AMF	Unknown	Unknown	WHOLE	Unknown	Unknown
Tinker	12	AMF	Unknown	Unknown	WHOLE	Unknown	Unknown
Tinker	29	AMF	Unknown	Unknown	WHOLE	Unknown	Unknown
Tinker	35	AMF	Unknown	Unknown	WHOLE	Unknown	Unknown
Tinker	47	AMF	Unknown	Unknown	WHOLE	Unknown	Unknown
Tinker	68	AMF	Unknown	Unknown	WHOLE	Unknown	Unknown
Tinker	79	AMF	Unknown	Unknown	WHOLE	Unknown	Unknown
Tinker	82	AMF	Unknown	Spread to 2	WHOLE	Unknown	Unknown
Tinker	92	AMF	Distance decay	Unknown	WHOLE	Unknown	Unknown
Tinker	263	AMF	Unknown	Unknown	WHOLE	Unknown	Unknown
Tinker	477	AMF	Unknown	Unknown	WHOLE	Unknown	Unknown
Chanute	3	AMF	Unknown	No spread	WHOLE	Unknown	No spread
Chanute	7	AMF	Spread to 2	No spread	WHOLE	Unknown	No spread
Chanute	8	AMF	Unknown	No spread	WHOLE	Unknown	No spread
Chanute	9	AMF	Spread to 2	Spread to 2	WHOLE	Unknown	Unknown
Chanute	10	AMF	Unknown	Unknown	WHOLE	Spread to 2	Unknown
Chanute	12	AMF	Distance decay	Spread to 0.5	WHOLE	Spread to 0.5	Unknown
Chanute	16	AMF	Distance decay	No spread	WHOLE	Spread to 2	No spread
Chanute	22	AMF	Unknown	No spread	WHOLE	Unknown	No spread
Chanute	24	AMF	Unknown	No spread	WHOLE	Unknown	No spread
Chanute	25	AMF	No spread	No spread	WHOLE	No spread	No spread
Chanute	26	AMF	Unknown	No spread	WHOLE	Distance decay	No spread
Chanute	29	AMF	Spread to 0.5	No spread	WHOLE	Spread to 2	Spread to 0.5
Chanute	32	AMF	Distance decay	No spread	WHOLE	No spread	No spread
Chanute	36	AMF	Unknown	No spread	WHOLE	Spread to 2	Distance decay
Chanute	39	AMF	Unknown	No spread	WHOLE	Unknown	No spread
Chanute	42	AMF	Unknown	No spread	WHOLE	Unknown	No spread
Chanute	44	AMF	Unknown	No spread	WHOLE	Unknown	No spread
Chanute	53	AMF	Unknown	No spread	WHOLE	Unknown	Spread to 0.5
Chanute	58	AMF	Unknown	No spread	WHOLE	Unknown	No spread
Chanute	60	AMF	Unknown	No spread	WHOLE	Unknown	No spread
Chanute	61	AMF	Unknown	No spread	WHOLE	Spread to 2	No spread
Chanute	68	AMF	Unknown	No spread	WHOLE	Spread to 0.5	Distance decay
Chanute	70	AMF	Unknown	Unknown	WHOLE	Unknown	Spread to 0.5
Chanute	71	AMF	No spread	No spread	WHOLE	No spread	No spread
Chanute	72	AMF	Unknown	No spread	WHOLE	Unknown	No spread
Chanute	74	AMF	Unknown	No spread	WHOLE	Spread to 2	Spread to 0.5
Chanute	78	AMF	No spread	No spread	WHOLE	No spread	No spread
Chanute	82	AMF	Unknown	No spread	WHOLE	Unknown	No spread
Chanute	89	AMF	No spread	Unknown	WHOLE	No spread	No spread
Chanute	93	AMF	Unknown	No spread	WHOLE	Spread to 2	Distance decay
Chanute	103	AMF	Unknown	No spread	WHOLE	No spread	No spread
Chanute	106	AMF	Unknown	No spread	WHOLE	Spread to 2	Spread to 0.5
Chanute	134	AMF	Unknown	Unknown	WHOLE	Spread to 2	No spread
Chanute	162	AMF	Unknown	Unknown	WHOLE	Unknown	Unknown

Chanute	171	AMF	Unknown	No spread	WHOLE	Unknown	No spread
Chanute	197	AMF	Spread to 0.5	No spread	WHOLE	Distance decay	No spread
Chanute	207	AMF	Distance decay	Unknown	WHOLE	No spread	Unknown
Chanute	229	AMF	Unknown	Unknown	WHOLE	No spread	Unknown
Chanute	332	AMF	Unknown	Unknown	WHOLE	Unknown	Unknown
Chanute	436	AMF	Distance decay	Unknown	WHOLE	Distance decay	Unknown
Chanute	548	AMF	Unknown	Unknown	WHOLE	Unknown	Unknown
Chanute	570	AMF	Distance decay	Unknown	WHOLE	Unknown	Unknown
Chanute	794	AMF	Distance decay	Unknown	WHOLE	No spread	Unknown
Chanute	869	AMF	No spread	Distance decay	WHOLE	Distance decay	Unknown
Ft. Riley *B. bladhii*	26	AMF	Unknown	Unknown	WHOLE	Unknown	Unknown
Ft. Riley *B. bladhii*	30	AMF	Distance decay	Spread to 2	WHOLE	Distance decay	Distance decay
Ft. Riley *B. bladhii*	31	AMF	Unknown	Unknown	WHOLE	Unknown	Unknown
Ft. Riley *B. bladhii*	32	AMF	Unknown	Unknown	WHOLE	Unknown	Unknown
Ft. Riley *B. bladhii*	48	AMF	Distance decay	Unknown	WHOLE	Unknown	Unknown
Ft. Riley *B. bladhii*	49	AMF	Unknown	Unknown	WHOLE	No spread	Unknown
Ft. Riley *B. bladhii*	50	AMF	Unknown	Unknown	WHOLE	Unknown	Distance decay
Ft. Riley *B. bladhii*	60	AMF	Unknown	Unknown	WHOLE	Unknown	Unknown
Ft. Riley *B. bladhii*	70	AMF	Unknown	Unknown	WHOLE	Spread to 0.5	Unknown
Ft. Riley *B. bladhii*	74	AMF	Unknown	Unknown	WHOLE	Unknown	Unknown
Ft. Riley *B. bladhii*	76	AMF	Unknown	Unknown	WHOLE	Spread to 0.5	Unknown
Ft. Riley *B. bladhii*	79	AMF	Unknown	Unknown	WHOLE	Spread to 0.5	Unknown
Ft. Riley *B. bladhii*	91	AMF	Unknown	Unknown	WHOLE	Unknown	Unknown
Ft. Riley *B. bladhii*	93	AMF	Distance decay	Unknown	WHOLE	Unknown	Unknown
Ft. Riley *B. bladhii*	105	AMF	Unknown	Unknown	WHOLE	Unknown	Unknown
Ft. Riley *B. bladhii*	111	AMF	Unknown	Unknown	WHOLE	Distance decay	Unknown
Ft. Riley *B. bladhii*	116	AMF	Unknown	Unknown	WHOLE	Unknown	Unknown
Ft. Riley *B. bladhii*	118	AMF	Unknown	Unknown	WHOLE	Unknown	Unknown
Ft. Riley *B. bladhii*	121	AMF	Unknown	Unknown	WHOLE	Unknown	Unknown
Ft. Riley *B. bladhii*	123	AMF	Unknown	Unknown	WHOLE	Unknown	Unknown
Ft. Riley *B. bladhii*	130	AMF	Unknown	Unknown	WHOLE	Unknown	Unknown
Ft. Riley *B. bladhii*	135	AMF	Unknown	Unknown	WHOLE	Unknown	Unknown
Ft. Riley *B. bladhii*	136	AMF	Unknown	Distance decay	WHOLE	Distance decay	Distance decay
Ft. Riley *B. bladhii*	152	AMF	Unknown	Distance decay	WHOLE	Unknown	Distance decay
Ft. Riley *B. bladhii*	206	AMF	Unknown	Distance decay	WHOLE	Spread to 0.5	Distance decay
Ft. Riley *B. bladhii*	212	AMF	Unknown	Unknown	WHOLE	No spread	Unknown
Ft. Riley *B. bladhii*	239	AMF	Unknown	Unknown	WHOLE	Unknown	Distance decay
Ft. Riley *B. bladhii*	259	AMF	Unknown	Unknown	WHOLE	Unknown	Unknown
Ft. Riley *B. bladhii*	268	AMF	Unknown	Unknown	WHOLE	Unknown	Unknown
Ft. Riley *B. bladhii*	282	AMF	Unknown	Unknown	WHOLE	Unknown	Unknown
Ft. Riley *B. bladhii*	289	AMF	Unknown	Unknown	WHOLE	Unknown	Unknown
Ft. Riley *B. bladhii*	292	AMF	Unknown	No spread	WHOLE	Unknown	Unknown

Ft. Riley *B. bladhii*	314	AMF	Unknown	Unknown	WHOLE	Unknown	Distance decay
Ft. Riley *B. bladhii*	317	AMF	Unknown	Unknown	WHOLE	Unknown	Unknown
Ft. Riley *B. bladhii*	347	AMF	Unknown	Unknown	WHOLE	Unknown	Unknown
Ft. Riley *B. bladhii*	385	AMF	Unknown	Unknown	WHOLE	Unknown	Unknown
Ft. Riley *B. bladhii*	387	AMF	Unknown	Unknown	WHOLE	No spread	Unknown
Ft. Riley *B. bladhii*	401	AMF	Unknown	Unknown	WHOLE	Unknown	Unknown
Ft. Riley *B. bladhii*	409	AMF	Unknown	Distance decay	WHOLE	Unknown	Distance decay
Ft. Riley *B. bladhii*	447	AMF	Unknown	Unknown	WHOLE	Unknown	Unknown
Ft. Riley *B. bladhii*	450	AMF	Unknown	Unknown	WHOLE	Unknown	Unknown
Ft. Riley *B. bladhii*	503	AMF	Unknown	Unknown	WHOLE	Unknown	Unknown
Ft. Riley *B. inermis*	26	AMF	Unknown	Unknown	WHOLE	Unknown	Unknown
Ft. Riley *B. inermis*	30	AMF	Unknown	Unknown	WHOLE	Unknown	Distance decay
Ft. Riley *B. inermis*	31	AMF	Distance decay	Unknown	WHOLE	Distance decay	Unknown
Ft. Riley *B. inermis*	32	AMF	Unknown	Unknown	WHOLE	Distance decay	Unknown
Ft. Riley *B. inermis*	48	AMF	Distance decay	Unknown	WHOLE	Spread to 0.5	Unknown
Ft. Riley *B. inermis*	49	AMF	Distance decay	No spread	WHOLE	Distance decay	Unknown
Ft. Riley *B. inermis*	50	AMF	Distance decay	Unknown	WHOLE	Distance decay	Unknown
Ft. Riley *B. inermis*	60	AMF	No spread	Unknown	WHOLE	Unknown	Spread to 2
Ft. Riley *B. inermis*	70	AMF	Unknown	Unknown	WHOLE	Unknown	Unknown
Ft. Riley *B. inermis*	74	AMF	Unknown	Unknown	WHOLE	Unknown	No spread
Ft. Riley *B. inermis*	76	AMF	Unknown	Unknown	WHOLE	Unknown	Unknown
Ft. Riley *B. inermis*	79	AMF	Unknown	Unknown	WHOLE	Unknown	Unknown
Ft. Riley *B. inermis*	91	AMF	Unknown	Unknown	WHOLE	Spread to 2	Unknown
Ft. Riley *B. inermis*	93	AMF	Unknown	Unknown	WHOLE	Unknown	Unknown
Ft. Riley *B. inermis*	105	AMF	Unknown	Unknown	WHOLE	Unknown	Unknown
Ft. Riley *B. inermis*	111	AMF	No spread	Unknown	WHOLE	Distance decay	Unknown
Ft. Riley *B. inermis*	116	AMF	Unknown	Unknown	WHOLE	Unknown	Unknown
Ft. Riley *B. inermis*	118	AMF	Unknown	Unknown	WHOLE	Unknown	Unknown
Ft. Riley *B. inermis*	121	AMF	Unknown	Unknown	WHOLE	Unknown	Unknown
Ft. Riley *B. inermis*	123	AMF	Unknown	Unknown	WHOLE	Unknown	Unknown
Ft. Riley *B. inermis*	130	AMF	Unknown	Unknown	WHOLE	Unknown	Unknown
Ft. Riley *B. inermis*	135	AMF	Unknown	Spread to 2	WHOLE	Unknown	Unknown
Ft. Riley *B. inermis*	136	AMF	Unknown	Unknown	WHOLE	Unknown	Unknown
Ft. Riley *B. inermis*	152	AMF	Unknown	Unknown	WHOLE	Unknown	Unknown
Ft. Riley *B. inermis*	206	AMF	Unknown	Unknown	WHOLE	Unknown	Unknown
Ft. Riley *B. inermis*	212	AMF	Unknown	Unknown	WHOLE	Unknown	Unknown
Ft. Riley *B. inermis*	239	AMF	Unknown	Unknown	WHOLE	Unknown	Unknown
Ft. Riley *B. inermis*	259	AMF	Unknown	Unknown	WHOLE	Unknown	Unknown
Ft. Riley *B. inermis*	268	AMF	Spread to 2	Unknown	WHOLE	Distance decay	Unknown
Ft. Riley *B. inermis*	282	AMF	Spread to 2	Unknown	WHOLE	Unknown	Unknown
Ft. Riley *B. inermis*	289	AMF	Unknown	Unknown	WHOLE	Unknown	Unknown
Ft. Riley *B. inermis*	292	AMF	Unknown	Unknown	WHOLE	No spread	No spread

Ft. Riley *B. inermis*	314	AMF	Unknown	Unknown	WHOLE	Unknown	No spread
Ft. Riley *B. inermis*	317	AMF	Unknown	Unknown	WHOLE	Unknown	Unknown
Ft. Riley *B. inermis*	347	AMF	Unknown	Unknown	WHOLE	Unknown	Unknown
Ft. Riley *B. inermis*	385	AMF	Unknown	Unknown	WHOLE	Unknown	Unknown
Ft. Riley *B. inermis*	387	AMF	Unknown	Unknown	WHOLE	Unknown	Unknown
Ft. Riley *B. inermis*	401	AMF	Spread to 2	Unknown	WHOLE	Unknown	Unknown
Ft. Riley *B. inermis*	409	AMF	Unknown	Distance decay	WHOLE	Unknown	Distance decay
Ft. Riley *B. inermis*	447	AMF	Unknown	Unknown	WHOLE	Unknown	Unknown
Ft. Riley *B. inermis*	450	AMF	Unknown	Unknown	WHOLE	Unknown	Unknown
Ft. Riley *B. inermis*	503	AMF	Unknown	Unknown	WHOLE	Unknown	Unknown

Table S4. The effects of AM fungal family and year of experiment on spread of AM fungal taxa at Chanute.

Effect	*df*	F	*p*
family	1, 35	3.09	0.088
year	1, 35	12.38	**0.001**

Random	Estimate	SE
OTU x family x year	0.67	0.09

Table S5. ANOVA table displaying the effects of site, soil treatment (inocula), distance from nurse plant row, year, and all possible interactions on relative arbuscular mycorrhizal fungal biomass measured through neutral lipid fatty acid analyses (NLFAs).

Source	*df*	*F*	*P*
site	3	168.15	**< 0.0001**
soil treatment	2	1.87	0.13
distance	2	11.23	**0.004**
year	1	153.02	**< 0.0001**
site x soil treatment	6	1.02	0.41
site x distance	6	28.08	**< 0.0001**
soil treatment x distance	4	0.09	0.42
site x year	3	18.19	**< 0.0001**
soil treatment x year	2	4.08	**0.01**
distance x year	2	3.00	**0.05**
site x soil treatment x distance	10	1.22	0.27
site x soil treatment x year	6	1.74	0.12
site x distance x year	5	2.80	**< 0.03**
soil treatment x distance x year	4	0.22	0.92
site x soil treatment x distance x year	8	0.63	0.63

Table S6. ANOVA table displaying the effects of site, soil treatment (inocula), distance from nurse plant row, year, and all possible interactions on relative arbuscular mycorrhizal fungal biomass measured through phospholipid fatty acid analyses (PLFAs).

Source	*df*	*F*	*P*
site	3	225.99	**< 0.0001**
soil treatment	2	1.19	0.30
distance	2	5.31	**0.004**
year	1	35.70	**< 0.0001**
site x soil treatment	6	1.21	0.30
site x distance	6	3.13	**0.005**
soil treatment x distance	4	0.57	0.68
site x year	3	12.18	**< 0.0001**
soil treatment x year	2	0.52	0.59
distance x year	2	25.67	**< 0.0001**
site x soil treatment x distance	10	1.74	0.07
site x soil treatment x year	6	0.89	0.49
site x distance x year	5	38.50	**< 0.0001**
soil treatment x distance x year	4	0.20	0.94
site x soil treatment m x distance x year	8	1.16	0.32

Figure S1. Arbuscular mycorrhizal fungal biomass by distance for each site and year measured through PLFAs.

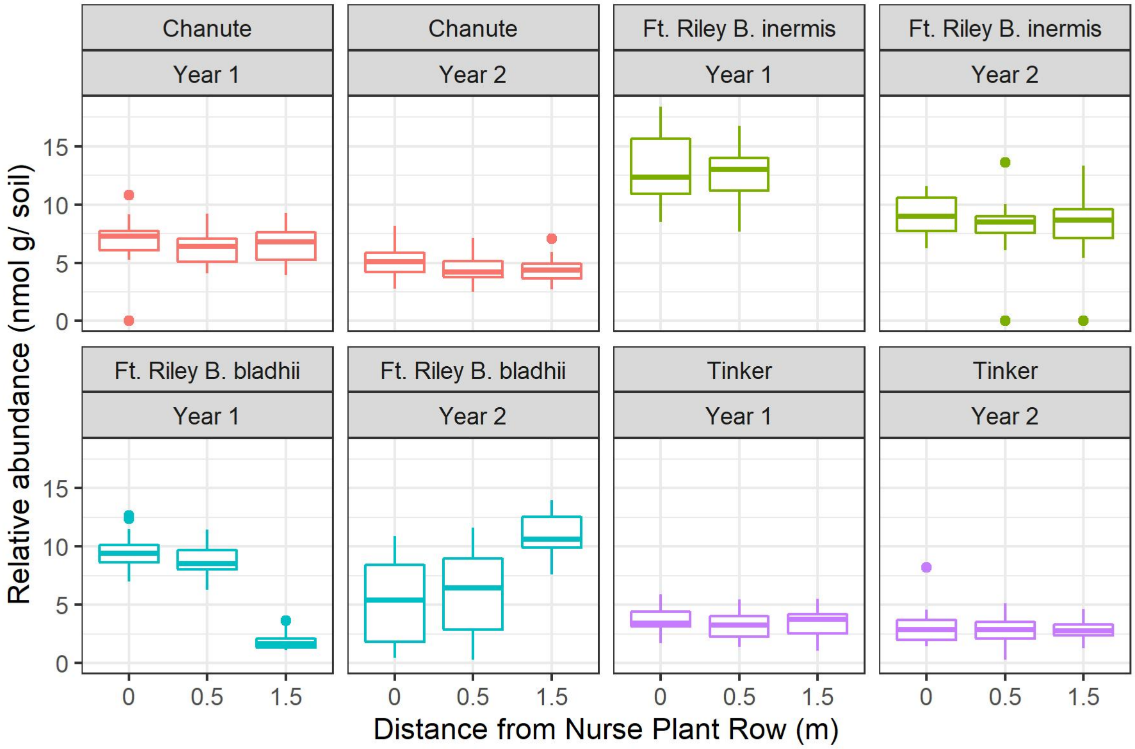

Figure S2. Arbuscular mycorrhizal fungal biomass (measured through NLFA) present at 1.5 m from the nurse plant row on the bridge and island sides at Tinker. Soil treatment: Sterile indicates non-inoculated control; AMF indicates nurse plants inoculated with AMF cultures; Whole indicates nurse plants inoculated with whole prairie soil.

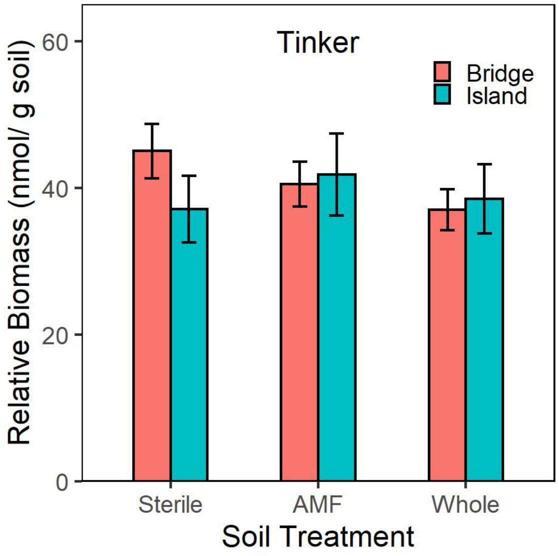

Figure S3. Arbuscular mycorrhizal fungal biomass (measured through PLFA) present at 1.5 m from the nurse plant row on the bridge and island sides at Tinker. Soil treatment: Sterile indicates non-inoculated control; AMF indicates nurse plants inoculated with AMF cultures; Whole indicates nurse plants inoculated with whole prairie soil.

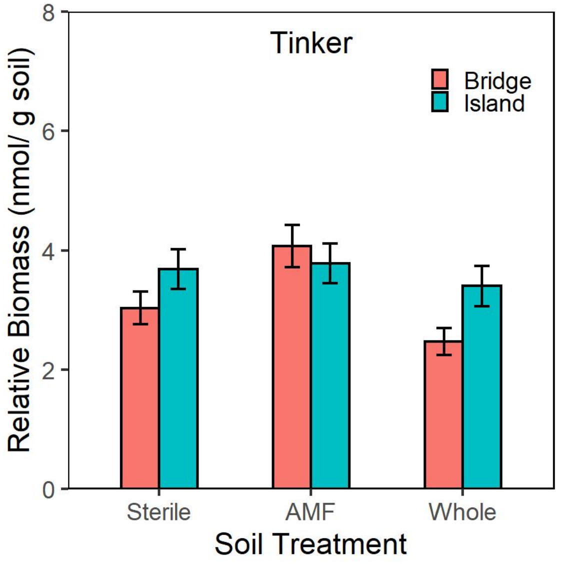

Figure S4. Arbuscular mycorrhizal fungal biomass (measured through NLFA) present at 1.5 m from the nurse plant row on the bridge and island sides at Chanute. Soil treatment: AMF indicates nurse plants inoculated with AMF cultures; Whole indicates nurse plants inoculated with whole prairie soil.

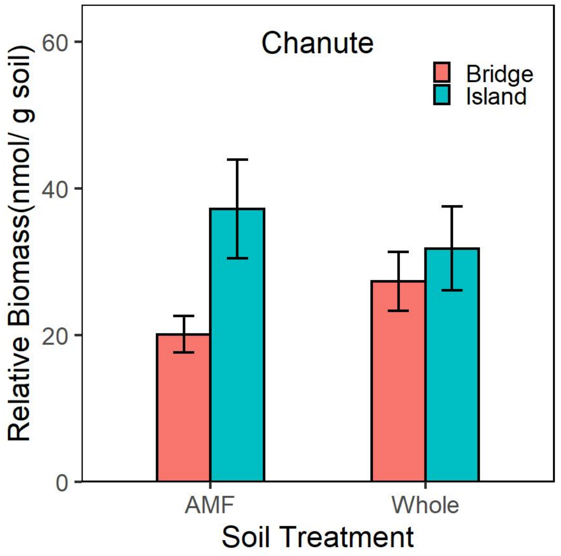

Figure S5. Arbuscular mycorrhizal fungal biomass (measured through PLFA) present at 1.5 m from the nurse plant row on the bridge and island sides at Chanute. Soil treatment: AMF indicates nurse plants inoculated with AMF cultures; Whole indicates nurse plants inoculated with whole prairie soil.

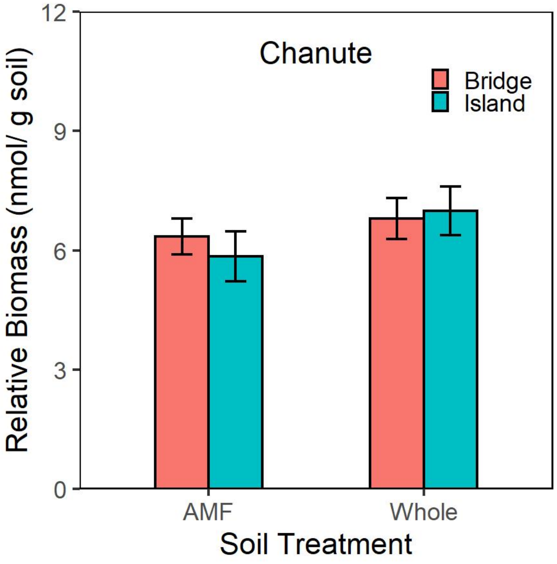

Figure S6. Plant community a) richness and b) diversity increases with time, except for in the third year at the Ft. Riley *B. baldhii* site, which decreases in the third year.

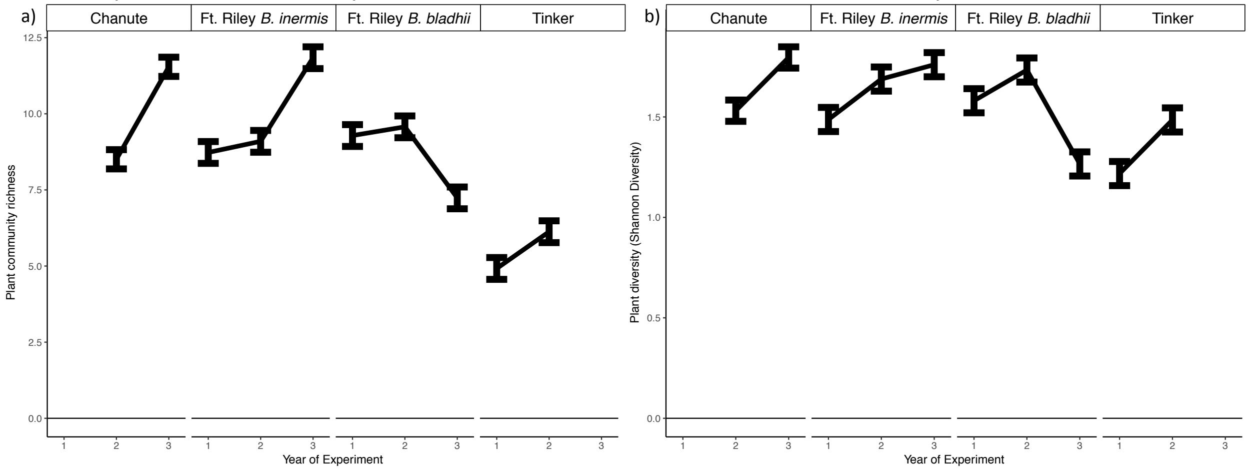

Figure S7. Diversity increased differently across years in different arbuscular mycorrhizal fungal inoculum treatments, specifically, the AMF treatment showing higher diversity in the third year at Ft. Riley *B. inermis* site.

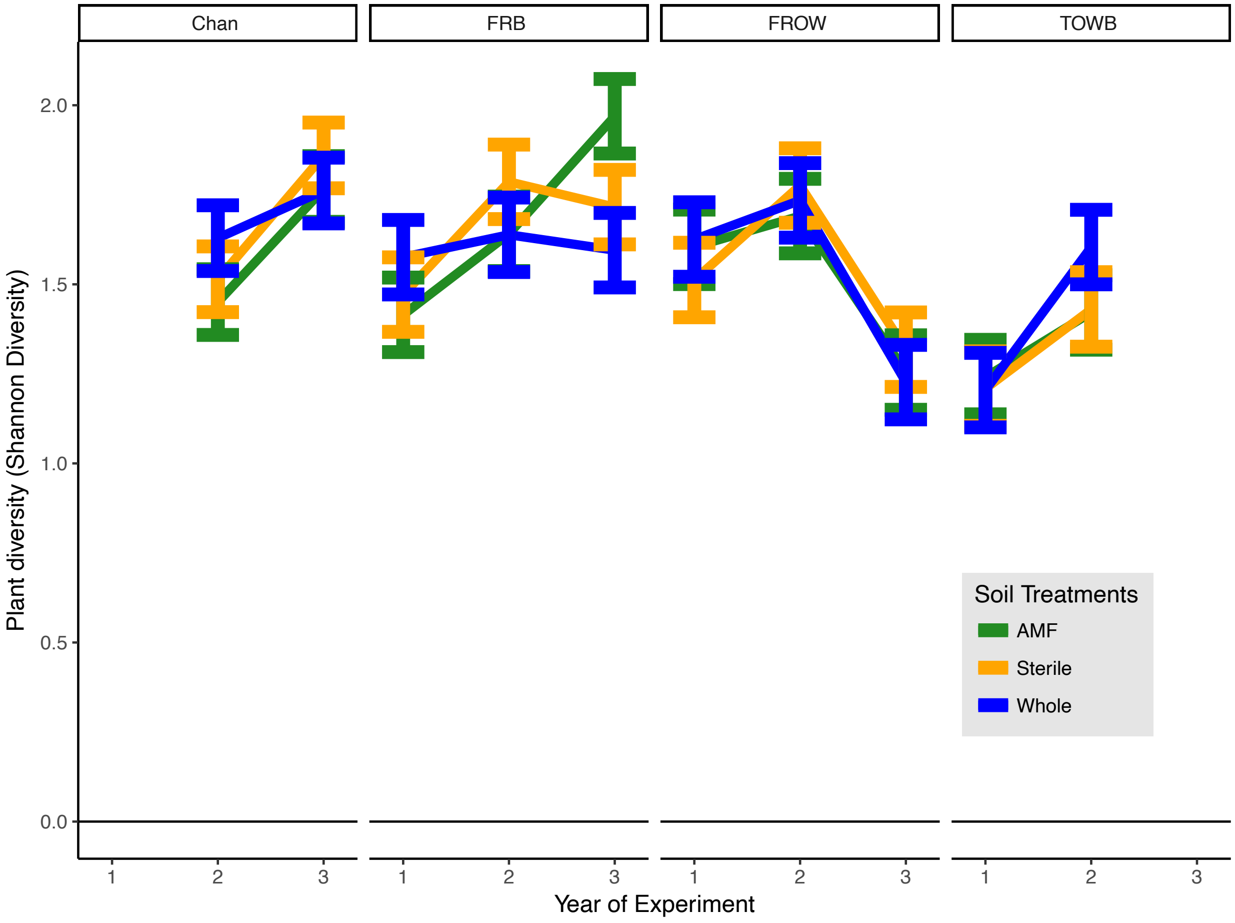

Figure S8. Plant evenness varied across arbuscular mycorrhizal fungal inoculum treatments in years, specifically plant evenness was greater in AMF plots compared to whole soil plots in Ft. Riley *B. inermis* site in the third year.

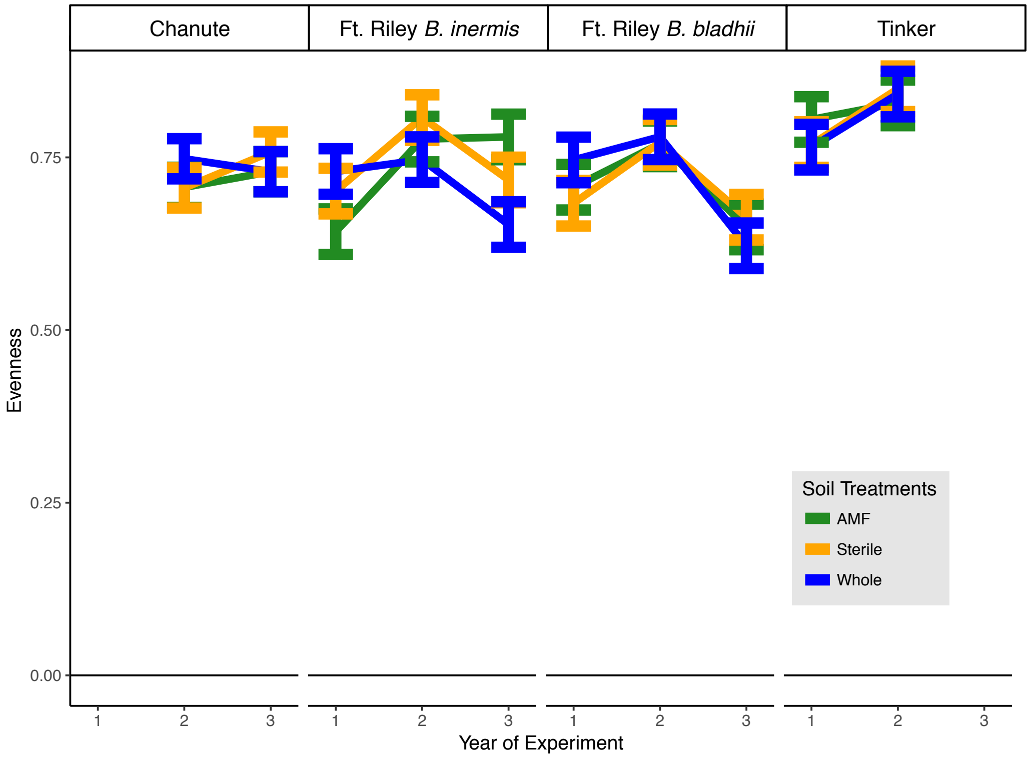

Figure S9. Ft. Riley *B. bladhii* site: a) plant richness in year one b) plant evenness in year two c) plant richness and d) plant diversity in year three across inoculum treatment and distance from nurse plant row, e) Plant evenness at Tinker site across soil treatment and distance all years.

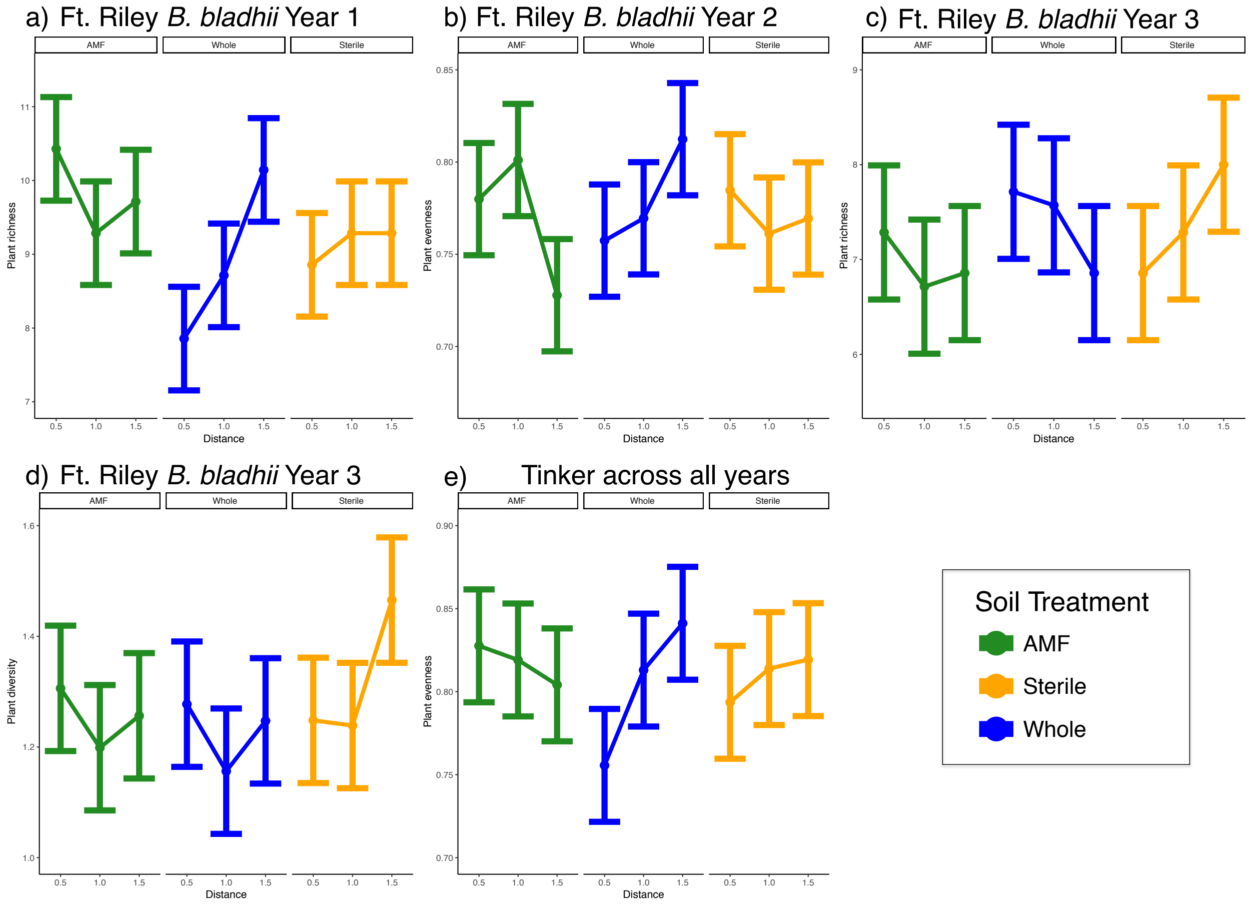

Figure S10. Plant community NMDS compostion graphs for a) Ft. Riley *B. bladhii* site in year one, b) Ft. Riley *B. bladhii* site in the second year, c) Tinker site across all years, and d) Ft. Riley *B. inermis* site in the third year.

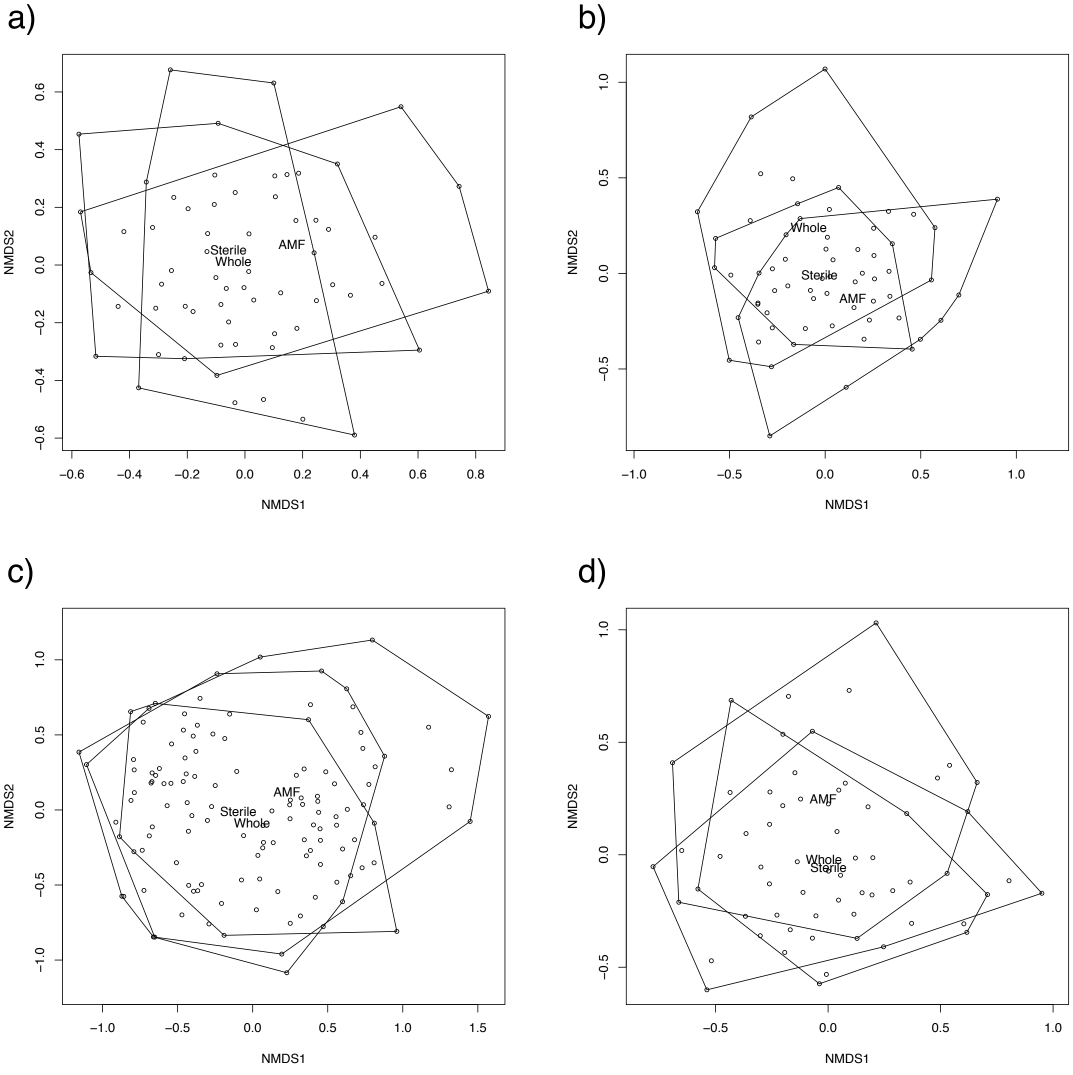

Figure S11. *Andropogon gerardii* test plants at Tinker were larger in arbuscular mycorrhizal fungal inoculated plots, and size decreased with distance from the nurse plant row across all plots.

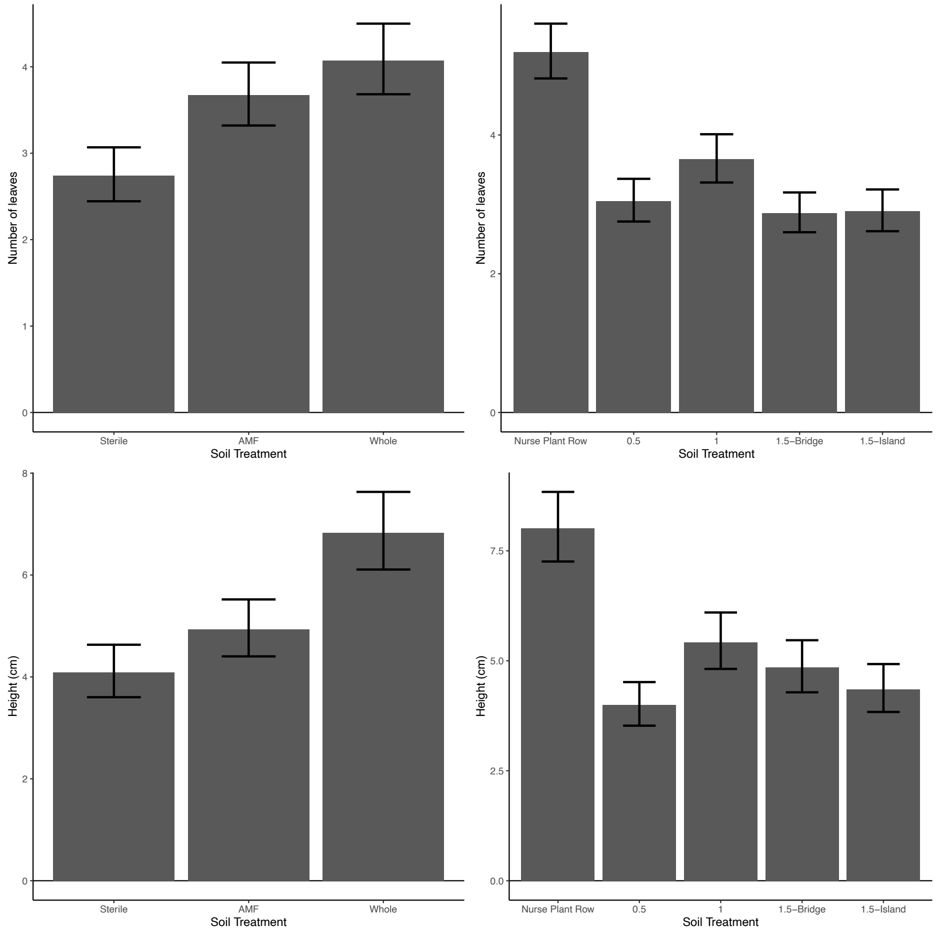

Supplement: Supplementary file 1 [file Data_Sheet_1.pdf]
